# Chitosan encompassed *Aniba rosaeodora* essential oil as innovative green candidate for antifungal and antiaflatoxigenic activity in millets with emphasis on cellular and its mode of action

**DOI:** 10.3389/fmicb.2022.970670

**Published:** 2022-08-09

**Authors:** Bijendra Kumar Singh, Anand Kumar Chaudhari, Somenath Das, Shikha Tiwari, Akash Maurya, Vipin Kumar Singh, Nawal Kishore Dubey

**Affiliations:** ^1^Laboratory of Herbal Pesticides, Centre of Advanced Study (CAS) in Botany, Institute of Science, Banaras Hindu University, Varanasi, India; ^2^Department of Botany, Government Girl’s Post Graduate College, Ghazipur, India; ^3^Department of Botany, Burdwan Raj College, Bardhaman, West Bengal, India

**Keywords:** *Aniba rosaeodora* essential oil, aflatoxin B_1_, *Setaria italica*, nanoencapsulation, safety profile, biodeterioration

## Abstract

The present study demonstrates first time investigation on encapsulation of *Aniba rosaeodora* essential oil into chitosan nanoemulsion (AREO-CsNe) with the aim of improvement of its antifungal, and aflatoxin B_1_ (AFB_1_) inhibitory performance in real food system. The GC–MS analysis of AREO revealed the presence of linalool (81.46%) as a major component. The successful encapsulation of EO into CsNe was confirmed through SEM, FTIR, and XRD analysis. The *in-vitro* release study showed the controlled release of AREO. AREO-CsNe caused complete inhibition of *Aspergillus flavus* (AFLHPSi-1) growth and AFB_1_ production at 0.8 and 0.6 μl/ml, respectively, which was far better than AREO (1.4 and 1.2 μl/ml, respectively). Impairment of ergosterol biosynthesis coupled with enhancement of cellular materials leakage confirmed plasma membrane as the possible antifungal target of both AREO and AREO-CsNe. Significant inhibition of methylglyoxal (AFB_1_ inducer) synthesis in AFLHPSi-1 cells by AREO and AREO-CsNe confirmed their novel antiaflatoxigenic mode of action. *In-silico* molecular docking studies revealed effective interaction of linalool with Ver-1 and Omt-A proteins, leading to inhibition of AFB_1_ biosynthesis. Further, AREO-CsNe showed enhanced antioxidant activity with IC_50_ values 3.792 and 1.706 μl/ml against DPPH^•^ and ABTS^•+^ radicals, respectively. In addition, AREO-CsNe caused 100% protection of stored millets (*Setaria italica* seeds) from AFB_1_ contamination and lipid peroxidation over a period of 1 year without compromising its sensory properties and exhibited high safety profile with LD_50_ value 9538.742 μl/kg body weight. Based on enhanced performance of AREO-CsNe over AREO, it can be recommended as a novel substitute of synthetic preservative for preservation of stored millets.

## Highlights

- Nanoencapsulation and characterization of AREO into chitosan nanoemulsion (CsNe).
- AREO nanoemulsion possesses improved antifungal and AFB_1_ inhibitory activities.
- Nanoemulsion exhibited strong *in-situ* preservative efficacy for stored *Setaria italica.*
- Nanoemulsion as protectant of millets from lipid oxidation with high safety profile.
- AREO nanoemulsion as green preservative for recommendation in food industry.

## Introduction

Millets are considered as major functional foods and currently receiving global attention as nutraceuticals due to high proteins, carbohydrate, and minerals along with their low glycemic index (Duodu and Dowell, 2019; Sruthi and Rao, 2021). Among different millets, *Setaria italica* (L.) P. Beauv. has strong ability to fulfil the minerals and micronutrients deficiency in human (Vetriventhan and Upadhyaya, 2019). The grains of this millet is well acknowledged in food nutrition due to abundant essential amino acids, proteins, and minerals such as potassium, calcium, zinc, phosphorous, zinc, and vitamin B (Bandyopadhyay et al., 2017a,b; Nadeem et al., 2018; Jaiswal et al., 2019).

However, during storage their grains are frequently contaminated by a number of storage fungi and related mycotoxins such as aflatoxins, ochratoxins, fumonisins, deoxynevalenol, and zearalenone which upon consumption can acute as well as chronic toxicity. Among different mycotoxins, aflatoxin B_1_ (AFB_1_) is of momentous concern owing to its proven carcinogenic, mutagenic, teratogenic, immunosuppressive, and nephrotoxic properties in both humans and animals (Chaudhari et al., 2019; Chibuzor-Onyema et al., 2021). Besides fungal and aflatoxin contamination, lipid peroxidation of stored millets is the second leading cause responsible for their qualitative deterioration (Ajiboye et al., 2017). The free radical oxygen species generated during lipid peroxidation has been also linked to be responsible for the generation of methylglyoxal (MG), substrate responsible for enhancing AFB_1_ in *Aspergillus flavus* culture (Chen et al., 2004).

To overcome these, various chemical preservatives have been widely applied; however, their indiscriminate use has led to the development of resistance races among fungal population, residual toxicity to non-target organisms, and environmental problems (Contigiani et al., 2018; OuYang et al., 2020). In this regard, the use of plant essential oils (EOs) as green chemicals is gaining cumulative interest because of their highly volatility that act as fumigant, biodegradability, safety and antimicrobial, insecticidal, and antioxidant activities (Isman, 2020; Das et al., 2021a; Yang et al., 2021a). Despite of having proven potential, the large-scale application of EOs especially in food matrix remains limited (Froiio et al., 2019; Ju et al., 2019). To overcome these limitations and to improve its wider practical applicability, their nanoencapsulation into suitable polymeric matrix has been recommended (Bagheri et al., 2021; Upadhyay et al., 2021). Among different polymers available, chitosan has received maximum interest due to its abundance, biodegradability, cost-effectiveness, green image, antimicrobial and antioxidant properties, and high encapsulation efficiency (Singh et al., 2021; Soltanzadeh et al., 2021).

*Aniba rosaeodora* Ducke (Family: Lauraceae), commonly known as rose wood, is a large perennial tree and grows abundantly in Amazon Forest (Sampaio et al., 2012; Maia and Mourão, 2016). The EO of *A. rosaeodora* (AREO) obtained from the Heart wood by steam distillation and displayed potential antimicrobial and antioxidant properties (Pimentel et al., 2018; Teles et al., 2021). However, reports pertaining to the antifungal and antiaflatoxigenic activity of AREO along with enhancement in overall bioefficacy after nanoencapsulation are still lacking.

Therefore, the main objective of this study was to synthesize and characterize AREO-based chitosan nanoemulsion (AREO-CsNe) and evaluate its preservative potential against fungal, AFB_1_ and lipid peroxidation mediated deterioration of stored millets. The study also elucidates the antifungal and antiaflatoxigenic mode of action of AREO by targeting plasma membrane and cellular methylglyoxal content, respectively in treated *A. flavus* strain. Finally, the safety profile was tested on mice model and *in-situ* study was conducted to explore the efficacy of nanoemulsion in real food system so as to recommend its possible large-scale application in food industry.

## Materials and methods

### Materials

Chitosan powder (deacetylation degree *>*85%), aqueous acetic acid, dichloromethane (DCM), sodium-tripolyphosphate (S-TPP), chloroform, Tween-20, Tween-80, acetic acid (99.99% purity), 1,1-diphenyl-2-picrylhydrazyl (DPPH), 2,2′-azino-bis (3-ethylbenzothiazoline-6-sulfonic acid; ABTS), 1,2-diaminobenzene (DAB), thiobarbituric acid (TBA), trichloroacetic acid (TCA), methylglyoxal, perchloric acid, sodium carbonate, and nutrient media (PDA and SMKY) were purchased from HiMedia laboratories, Mumbai, India. The EO of *A*. *rosaeodora* was procured from MRK naturals (99.90% purity), New Delhi, India.

### Fungal strain

Aflatoxigenic strain of *A. flavus* (AFLHPSi-1) and other food borne fungi *viz. A. niger*, *A*. *luchuensis*, *A*. *sydowii*, *A. minutus*, *A. chevalieri*, *A. humicola*, *A. fumigatus*, *A. nidulans*, *A. terreus*, *Fusarium graminearum*, *F*. *oxysporum*, *Penicillium citrinum*, *P. italicum*, and mycelia sterilia isolated during mycobiota analysis of different millet samples in our previous investigation (data unpublished) were used as test fungi. The fungal isolates isolated during the mycobiota analysis were identified with the help of standard manuals. The genus *Aspergillus* was identified by using taxonomic key of Raper and Fennel (1965), genus *Penicillium* was identified with the help of Manual of Pitt (1979), and other fungal isolates were identified using manual of soil fungi by Gilman and Joseph (1998).

### Isolation and characterisation of AREO

The AREO was obtained from the dried leaves of *A*. *roseodora* plant through conventional hydro-distillation in a Clevenger type apparatus following official method of European Pharmacopoeia (WorldCat, 2011). Chemical profiling of AREO was done through GC/MS (GC: Thermo scientific 1,300 GC and MS: Perkin Elmer Turbo mass Gold MA, United States) equipped with TG-5 capillary column (30 m × 0.25 mm ID × 0.25 μm thickness) and temperature of instrument was set from 60°C to 240°C with temperature rise at the rate of 5°C min^−1^. The split ratio was kept 1: 50, helium was used as carrier gas, transfer line and oven temperatures were set according to standard protocols. The identification of components was done by comparing their kovats retention indices (KRI) and mass fragmentation pattern with those available in the literature (Adams, 2007). The KRI values of different components were calculated by using the retention times (RT) of a homologous series of n-alkanes (C_9_–C_28_ hydrocarbons, Polyscience Corp. Niles IL) running in parallel with EO.

### Nanoencapsulation of AREO into chitosan matrix (AREO-CsNe)

AREO-loaded chitosan nanoemulsion was prepared through ionic gelation technique following Yoksan et al. (2010) with slight modifications. Briefly, 1.5% chitosan solution was prepared by overnight stirring in aqueous acetic acid (1%, *v/v*) solution followed by drop wise addition of Tween-80 as surfactant and stirring for 2 h at 45°C. Requisite amounts of AREO (0.06, 0.12, 0.18, 0.24, and 0.30 g) were separately dissolved in 4 ml dichloromethane (DCM) and added dropwise into chitosan solution during homogenization (12,600 rpm 10 min). Then, 0.4% solution of S-TPP was added in oil–water emulsion dropwise and stirred for 45 min. The prepared nanoparticles were collected through centrifugation (REMI compufuge CPR-4) at 13,000 × *g* for 10 min at 4°C and consequently washed thrice with 0.1% Tween-20 solution. Chitosan nanoemulsion (CsNe) was also prepared without addition of AREO by similar procedure. The emulsions were instantly sonicated by ultra-sonicator (Sonics Vibra Cell) for 8 min (1 s pulse on and 1 s pulse off) to obtain homogeneous suspension. Different ratios of chitosan to AREO (1: 0.0, 1: 0.2, 1: 0.4, 1: 0.6, 1: 0.8, and 1: 1) were prepared and assessed for loading in chitosan. Finally, the nanoemulsion was lyophilized (Christ, alpha D plus, Australia) at −54°C for 48 h and the obtained powdered nanoparticles were used for physico-chemical characterizations. All the biological experiments were performed by prepared nanoemulsion.

### Determination of loading capacity and encapsulation efficiency of AREO-CsNe

Briefly, 300 μl aliquot of AREO nanoemulsion was dissolved into 3 ml of hexane followed by gentle mixing and centrifugation at 13,000 × *g* for 10 min. Absorbance of collected supernatant was recorded at 239 nm by UV–Visible spectrophotometer. The amount of AREO present in supernatant was calculated from standard curve obtained in pure hexane (*y* = 0.0019 x − 0.0085, *R*^2^ = 0.9969). The amount of loaded AREO can be calculated by subtracting the amount of AREO present in supernatant from the amount of total AREO used. % Encapsulation efficiency (EE) and loading capacity (LC) were calculated from the following equations (1 and 2) of Hosseini et al. (2013).


(1) $$\begin{array}{c} \%EE=\left(\mathrm{Amount}\;\mathrm{of}\;\mathrm{loaded}\;\mathrm{AREO/Initial}\;\mathrm{of}\;\mathrm{AREO added}\right)\\ \;\times 100 \end{array}$$

(2) $$\begin{array}{c} \%LC=\left(\begin{array}{l} \mathrm{Amount}\;\mathrm{of}\;\mathrm{loaded}\;\mathrm{AREO}\;\mathrm{in}\;\mathrm{chitoson}\;\mathrm{nanoemulsion}\\ /\mathrm{weight}\;\mathrm{of nanoemulsion} \end{array}\right)\\ \;\times 100 \end{array}$$

### Physico-chemical characterization of AREO-CsNe

#### Scanning electron microscopic investigation

The morphological structures of synthesized nanoemulsions (CsNe and AREO-CsNe) were observed through scanning electron microscope (SEM; Evo-18 researcher, Zeiss, Germany). For this, 1 mg of powdered CsNe and AREO-CsNe were dissolved into 10 ml of double distilled water and sonicated for 4 min at 4°C. Thereafter, 10 μl aliquot of CsNe and AREO-CsNe was separately dropped on cover glass, spread to form thin film, and dried at room temperature. The dried films were then coated with gold and observed in SEM.

#### Fourier transform infrared spectroscopic analysis

Fourier transform infrared (FTIR) spectra of chitosan powder (CsP), AREO, lyophilized CsNe, and AREO-CsNe were recorded by Perkin Elmer IR spectrometer (United States). The powdered and liquid samples were mixed with KBr to form thin pellets and analysed under 64 scans at resolution of 4 cm^−1^ from 500 to 4,000 cm^−1^.

#### X-ray diffraction analysis

X-ray diffractometer (Bruker D8 Advance) was used to analyse the crystallographic profiles of CsP, CsNe, and AREO-CsNe. The measurement was done over the 2θ range from 5 to 50° with step angle of 0.02° min^−1^ and scanned speed of 5° min^−1^.

### *In-vitro* release profile of AREO-CsNe

The *in-vitro* release profile of AREO-CsNe was performed in phosphate buffer saline (PBS) mixed ethanol solution following the protocol of Das et al. (2020). Briefly, an aliquot of 500 μl AREO-CsNe was dissolved in 3 ml of PBS (pH 7.4) and absolute ethanol (3:2 *v*/*v*) and kept at room temperature. The ethanol was used for proper phase separation and breaking of AREO-CsNe emulsionic particles in the PBS system. Moreover, ethanol addition facilitates proper partitioning of emulsion phases and interphases outside the core material induced by reduction in interfacial tension and delivery of AREO (Das et al., 2021b). At specific time intervals (8 to 152 h), the sample was centrifuged at 13,000 × *g* and 1 ml of the supernatant was taken out for analysis followed by replacement of PBS and ethanol mixture to maintain the total volume. The amount of AREO released was determined at 239 nm using the standard calibration curve of AREO (*y* = 0.0238x + 0.0015; *R*^2^ = 0.9841) prepared in PBS and ethanol mixture. The percent cumulative release was calculated by equation (3).


(3) $$\begin{array}{l} \begin{array}{lll} \begin{array}{l} \%\;\mathrm{Cumulative}\;\mathrm{release}\;\\ \mathrm{of}\;\mathrm{AREO} \end{array} & = & \frac{\begin{array}{c} \mathrm{Cumulative}\;\mathrm{amount}\;\mathrm{of}\;\mathrm{AREO}\;\\ \mathrm{released}\;\mathrm{at}\;\mathrm{each time} \end{array}}{\begin{array}{c} \mathrm{Initial}\;\mathrm{amount}\;\mathrm{of}\;\mathrm{AREO}\;\\ \mathrm{loaded}\;\mathrm{in}\;\mathrm{the}\;\mathrm{sample} \end{array}}\times 100 \end{array} \end{array}$$

### Antifungal and antiaflatoxigenic efficacy of AREO and AREO-CsNe against AFLHPSi-1

Requisite concentrations of AREO (0.2–1.4 μl/ml) and AREO-CsNe (0.2–0.8 μl/ml) were added in 25 ml SMKY medium in conical flasks. Thereafter, each conical flask was inoculated with 25 μl spore suspension of AFLHPSi-1 (density = 10^6^ spores/mL) strain. Controls were prepared by addition of 500 μl of 0.5% Tween 20 for AREO and CsNe for AREO-CsNe. Thereafter, the conical flasks were kept at 27 ± 2°C in BOD incubator for 10 days. The minimum concentration of AREO and AREO-CsNe preventing the visible growth of test fungus (determined in terms of fungal mycelial fresh weight calculated by weighing the soaked filtered fungal mycelia after harvestation) was considered as minimum inhibitory concentrations (MICs).

The antiaflatoxigenic activity of AREO and AREO-CsNe was evaluated as minimum AFB_1_ inhibitory concentration (MAIC). For the quantification of AFB_1_, the contents (media and mycelia) of cultured isolates were filtered through Whatman no. 1 paper. AFB_1_ was extracted from the medium in separating funnel using 20 ml chloroform followed by evaporation on water bath (80°C) till complete dryness and residues were re-dissolved in 1 ml methanol. 50 μl aliquot of each sample was spotted on silica gel G plate and developed in a mixture consisting of toluene: isoamyl alcohol: methanol, 90:32:2 (*v*/*v*/*v*) and observed under UV transilluminator (Zenith, India) at 360 nm. The fluorescence of blue-coloured spots indicated the presence of AFB_1_. Spots were scrapped, dissolved in 5 ml cold methanol, and centrifuged at 5000 × *g* for 10 min. The absorbance of each sample was recorded at 360 nm using UV–visible spectrophotometry (UV-2600, Shimadzu, Japan) and amount of AFB_1_ was calculated using following equation (4).


(4) $$AFB1\;\mathrm{content}\left(\frac{\mathrm{\mu g}}{\mathrm{mL}}\right)=\frac{D\times M}{E\times L}\times 1000$$

Where, D = absorbance of samples, M = molecular weight of AFB_1_ (312), E = molar extinction coefficient of AFB_1_ (21,800), and L = path length (1 cm).

### Fungitoxic spectrum of AREO and AREO-CsNe

Fungitoxic spectrum of AREO and AREO-CsNe was determined against 14 different storage fungi (*A. niger*, *A*. *luchuensis*, *A*. *sydowii*, *A. minutus*, *A. chevalieri*, *A. humicola*, *A. fumigatus*, *A. nidulans*, *A. terreus*, *Fusarium graminearum*, *F*. *oxysporum*, *Penicillium citrinum*, *P. italicum*, and mycelia sterilia) using the poisoned food technique. The inhibition of fungal growth was recorded and % inhibition was calculated by following Equation (5):


(5) $$\%\;\mathrm{Growth inhibition}=\frac{\mathrm{dc}-dt}{\mathrm{dc}}\times 100$$

Where, dc = average colony diameter in control sets, and dt = average diameter of fungal colony in treatment sets.

### Biochemical and molecular docking concerning the mode of action of both AREO and AREO-CsNe

#### Effect on ergosterol biosynthesis

Effect of AREO and AREO-CsNe on plasma membrane ergosterol of AFLHPSi-1 cells was studied following the methods described by Das et al. (2022). Requisite amounts of AREO (0.2–1.4 μl/ml) and AREO-CsNe (0.2–0.8 μl/ml) were added to SMKY medium (25 ml) and inoculated with 25 μl spore suspension. After 7 days of incubation at 27 ± 2°C in BOD incubator, the fungal biomass was autoclaved, and then dipped in culture tube containing 5 ml of 25% alcoholic KOH solution and vortexed for 3 min. The mixture was then incubated on water bath for 2 h at 75°C, cooled, and 2 ml of distilled water and 5 ml of n-heptane were added, followed by vortex mixing for 3 min. After completion of incubation period (1 h), the upper n-heptane layer was scanned using UV–visible spectrophotometer between 230 to 300 nm and ergosterol contents of samples were calculated by the equation (6).

% Ergosterol = A (% ergosterol + % 24 (28) dehydroergosterol) – B (% 24 (28) dehydroergosterol)


(6) $$\begin{array}{l} \begin{array}{l} A\left(\%\;\mathrm{ergosterol}+\%\;24\left(28\right)\;\mathrm{dehydroergosterol}\right)\\ =\mathrm{Abs}.282/290)/\mathrm{pellet}\;\mathrm{weight} \end{array}\\ \begin{array}{l} B\left(\%\;24\left(28\right)\mathrm{dehydroergosterol}\right)\\ =\mathrm{Abs}.230/518/\mathrm{pellet}\;\mathrm{weight} \end{array} \end{array}$$

Where, 290 and 518 are the E values in % per cm for crystalline ergosterol and 24 (28) dehydroergosterol and pellet weight (g).

#### Determination of vital cellular ions and UV-absorbing material leakage

Estimation of ions leakage from AFLHPSi-1 cells treated with AREO and AREO-CsNe were performed following the protocol of Chaudhari et al. (2020b,c) with slight modifications. The samples were analysed for Ca^2+^, Mg^2+^ and K^+^ ions and 260, 280 nm absorbing materials leakage through atomic absorption spectrophotometry (AAnalyst 800, Perkin Elmer, United States) and UV–visible spectrophotometry, respectively.

#### Effect on methylglyoxal (MG) content of AFLHPSi-1 cells

Estimation of cellular MG in AFLHPSi-1 cells was done following the method of Upadhyay et al. (2018). In brief, 300 mg of harvested biomass of AFLHPSi-1 was placed in SMKY medium containing different concentrations of AREO (0.2 to 1.4 μl/ml) and AREO-CsNe (0.2 to 0.8 μl/ml). The control sets were prepared without AREO and AREO-CsNe. After overnight incubation in BOD incubator at 27 ± 2°C, mycelia was crushed in 3 ml of 0.5 M chilled perchloric acid and incubated in ice bath for 15 min. Thereafter, the incubated suspensions were centrifuged at 13,000 × *g* for 10 min and supernatant was neutralized by dropwise addition of saturated potassium carbonate solution. The samples were again centrifuged and obtained supernatant was used for cellular MG estimation. The standard curve of MG was prepared using different concentrations (10–100 μm) of pure MG.

### *In-silico* molecular docking of linalool with Ver-1 and Omt-A proteins

For molecular docking analysis, 3D structure of linalool (major component of AREO) was downloaded in SDF format from PUBCHEM online server. FASTA sequences of Ver-1 and Omt-A proteins were retrieved from Uniprot database and transferred to phyre 2 online server to develop 3D structures. Following this, molecular interaction of linalool with Ver-1 and Omt-A proteins was done in UCSF chimera software on the basis of number of hydrogen bond, and binding energy (kcal/mol) indices.

### Antioxidant activity evaluation of AREO and AREO-CsNe

#### DPPH^•+^ assay

For DPPH^•+^ assay, 0.004% methanolic solution of DPPH^•+^ was prepared and kept under dark condition overnight. Different volumes of AREO (5–50 μl/ml) and AREO-CsNe (5–50 μl/ml) were mixed in methanolic DPPH^•+^ solution to obtain different concentrations and incubated at room temperature for 30 min. Absorbance of the sample was measured at 517 nm and per cent free radical scavenging activity was calculated by following Equation (7):


(7) $$\%\;\mathrm{Inhibition}=\frac{\mathrm{Ablank}-\mathrm{Asample}}{\mathrm{Ablank}}\times 100$$

Where, A blank = absorbance of methanolic DPPH^•+^ solution;

A sample = absorbance of different concentrations of AREO and AREO-CsNe solutions.

#### ABTS assay

ABTS^•+^ scavenging activity of AREO and AREO-CsNe was estimated following the protocol of Re et al. (1999) with some modifications. Firstly, the ABTS^•+^ reaction mixture was prepared by mixing equal volumes of 7 mM stock solution of ABTS^•+^ with 140 mM of potassium persulphate and kept at room temperature under dark condition for 12–14 h. The prepared mixture was diluted with ethanol until absorbance of mixture maintained to 0.7 ± 0.05 nm. Thereafter, requisite volumes of AREO (2–20 μl/ml) and AREO-CsNe (2–20 μl/ml) were added into ABTS^•+^ mixture and after 6 min of incubation, absorbance of the samples was recorded at 734 nm. Per cent free radical scavenging activity was calculated by same Equation of DPPH assay.

### *In-situ* antifungal and AFB_1_ inhibitory activity of AREO and AREO-CsNe in *Setaria italica* (the model food system) seeds

*In-situ* efficacy of AREO and AREO-CsNe against AFLHPSi-1 was performed in *S. italica* seeds stored in airtight containers for 1 year (Chaudhari et al., 2020a). The experiment was performed in three different sets; in set 1, the millets seeds were fumigated with MIC doses of AREO (1.4 μl/ml) and AREO-CsNe (0.8 μl/ml). Set 2 contained millet seeds fumigated with 2 MIC doses of AREO (2.8 μl/ml) and AREO-CsNe (1.6 μl/ml). The set 3 was considered as control, where the millets seeds were not treated with AREO and/or AREO-CsNe. All the sets were stored at 25 ± 2°C and ~ 70% relative humidity for 1 year. After completion of storage periods, mycobiota analysis of seed samples was performed by serial dilution. For this, 1 g of grinded seed sample was dissolved into 9 ml of double distilled water and different dilutions were prepared. Thereafter, 1 ml aliquot of 10^−4^ dilution was inoculated into PDA medium in Petri plate followed by incubation at B.O.D incubator for 7 days at 25 ± 2°C. Fungal colonies in control and treatment sets were counted and percent protection was measured by the following equation.


$$\begin{array}{c} \%\;\mathrm{Fungal}\;\mathrm{protection}=\frac{\begin{array}{c} \mathrm{Number}\;\mathrm{of}\;\mathrm{fungle}\;\mathrm{colonies}\;\mathrm{in}\;\mathrm{control}\\ -\mathrm{Number}\;\mathrm{of}\;\mathrm{fungle}\;\mathrm{colonies}\;\mathrm{in}\;\mathrm{treatment} \end{array}}{\mathrm{Number}\;\mathrm{of}\;\mathrm{fungle}\;\mathrm{colonies}\;\mathrm{in}\;\mathrm{treatment}}\\ \;\times 100 \end{array}$$

The seed samples were processed and analysed for AFB_1_ determination through high performance liquid chromatography (HPLC). In brief, ten grams of finally grounded samples were mixed with 10 ml mixture of methanol and distilled water (8:10 *v*/*v*), placed on mechanical shaker for 30 min, and centrifuged at 5000 × *g* for 10 min. 4 ml of supernatant was mixed with 300 μl chloroform and 6 ml water containing 3% KBr (0.18 g KBr dissolved in 6 ml of water) and again centrifuged. The settled phase was collected, evaporated on water bath, and then dissolved in 500 μl of HPLC grade methanol. 50 μl of sample was injected to C^−18^ reverse phase column using the mobile phase of methanol, acetonitrile and distilled water (17:19:64 *v*/*v*/*v*). AFB_1_ content was expressed as μg/kg of *S. italica* seeds.

### Lipid peroxidation estimation in stored *Setaria italica* seeds

Lipid peroxidation in *S. italica* seeds treated with AREO and AREO-CsNe (MIC and 2 MIC doses respectively) were estimated in terms of malondialdehyde (MDA) content following Das et al. (2020) with minor modifications. 0.5 g grinded millet samples were mixed with 4 ml of reaction mixture containing 15% TCA, 0.375% TBA, and 0.25 N HCl followed by heating on water bath (75°C) for 10 min. Thereafter, the mixture was centrifuged at 10,000 × *g* for 10 min. The absorbance of the obtained supernatant was determined at 532 and 600 nm, respectively. The MDA content was calculated using the molar extinction coefficient 0.155 μm^−1^ cm^−1^ and expressed in terms of μm/gFW of *S. italica* seeds.

### Safety profile assessment of AREO and AREO-CsNe

The safety profile assessment of AREO and AREO-CsNe was carried out on male mice (*Mus musculus* L., average weight 35 g and 3 months old). The mice were obtained from the Department of Zoology, Banaras Hindu University, Varanasi and prior to experiment, they were acclimatized for 7 days under laboratory conditions. The permission from Animal care and Ethical committee of the University was taken prior to practicing acute oral toxicity study on mice. The stock solution of Tween-20 and distilled water (1:1) was prepared and different concentrations (100–500 μl/ml) of AREO and AREO-CsNe were orally administered to mice with the help of micropipette catheter. For control sets, equal amounts of Tween-20 and water (1:1) mixture were given. All groups of mice were observed periodically between 4 to 24 h, and at the end of period. Dead mice number was counted for the evaluation of LD_50_ values by applying Probit analysis (Finney, 1971).

### Phytotoxicity assessment of AREO and AREO-CsNe on stored *Setaria italica* seeds

For phytotoxicity assay, both the untreated and treated seeds were collected and rinsed with distilled water and placed in Petri plates containing moistened blotting papers at the bottom. At the regular time intervals (1–7 days), the lengths of plumules and radicles were recorded by centimetre scale.

### Sensory profile evaluation of AREO and AREO-CsNe in *Setaria italica* seeds

Sensorial analysis of *S. italica* seeds treated with AREO and AREO-CsNe at MIC and 2 MIC doses were carried out by a panel of 10 unaware trained peoples of both genders and they know the taste of millet because of their consuming experiences. Sensory analysis of seeds was done by the 5-point hedonic scale (5 = extremely acceptable, 4 = slightly acceptable, 3 = moderately acceptable, 2 = acceptable, and 1 = not acceptable) following the protocol of Chaudhari et al. (2020b). The parameters tested were colour, texture, odour, mouth feel, and overall acceptability. The samples were cooked in boiling water for half an hour without the use of pressure cooking, given to the participants in a transparent plate and coded with two arbitrary digits.

### Statistical analysis

Each experiment was conducted a minimum of three times, and each analysis was carried out in triplicate. The experimental data were subjected to one way analysis of variance (ANOVA), and significant differences between means were evaluated by Tukey’s B multiple range test (SPSS 25.0.) and value *p* < 0.05 was considered statistically significant.

## Results and discussion

### Chemical characterization of AREO

Chemical characterization of AREO exhibited the presence of 9 different components comprising 95.91% of total essential oil (Table 1). Linalool (81.46%) and α-Terpineol (7.4%) were observed as major components of AREO. The result of the present investigation is in agreement with the report of Teles et al. (2021) indicating linalool as major ingredient of AREO. Variations in the per cent composition of EOs are ascribed due to differences in extraction techniques, plant parts utilised for extraction of EO, time of harvesting, age of plant, thereby, affecting the chemical composition of AREO (Nattudurai et al., 2017).

**Table 1:** GC-MS of *Aniba rosaeodora* EO.

S. No.	Components	RT (min)	Percent composition	KRI (Adams, 2007)	KRI (calculated)
1	Linalool oxide	6.03	1.56	1,005	1,002
2	**Linalool** ^*^	**7.32**	**81.46**	**1,095**	**1,091**
3	Dihydrolinalool	7.98	1.1	1,131	1,128
4	α-Terpineol	10.94	7.4	1,186	1,181
5	γ-Terpineol	11.07	0.93	1,199	1,196
6	α-Gurjunene	14.62	1.36	1,409	1,407
7	β-Gurjunene	15.57	0.46	1,431	1,429
8	α-Caryophyllene	16.48	0.64	1,464	1,459
9	α-Longipinene	17.84	0.99	1,350	1,347
	**Total**		**95.91**		

* Major component in bold.

### Preparation of AREO loaded chitosan nanoemulsion (AREO-CsNe)

AREO loaded chitosan nanoemulsion (AREO-CsNe) was prepared through ionic gelation method. Most notably, chitosan and EO are kept under GRAS category, suggesting suitability for practical application in food systems (Alehosseini et al., 2021; Hu and Luo, 2021). AREO-CsNe complex is stabilized by interaction between phosphate groups of S-TPP and amine group of chitosan under slightly acidic condition, producing the stable nanoemulsion (Das et al., 2020). Similar method has been used and for the preparation of clove EO loaded chitosan nanoparticles and allspice EO loaded chitosan nanoemulsion by others (Hadidi et al., 2020; Chaudhari et al., 2022a). Using this method, the authors obtained good encapsulation efficiency and loading capacity for the respective EOs that may be useful for long time application as food preservative.

### Determination of loading capacity (LC) and encapsulation efficiency (EE)

The LC values of AREO-CsNe ranged between 0.38–2.48%, while EE was found in the range of 70.24–94.95% (Table 2). LC represents the concentration of EO in fixed amount of chitosan nanoemulsion, whereas EE represents the concentration of nanoencapsulated EO evaluated over the initial amount taken and was maximum at 1:1 chitosan to AREO ratio. Higher EE value strengthens the nanoencapsulated EOs against the oxidative damages and degradation under storage conditions (Timilsena et al., 2016). Similar trends of increasing EE and LC were reported by Singh et al. (2019) and Upadhyay et al. (2021) during encapsulating *Ocimum sanctum* and *Cananga odorata* EOs, respectively in chitosan nanoemulsion.

**Table 2:** Effect of different concentrations of AREO on encapsulation efficiency and loading capacity.

Chitosan: AREO (w/v)	% Encapsulation efficiency	% Loading capacity
1:0	0.000 ± 0.000^a^	0.000 ± 0.000^a^
1:0.2	70.208 ± 2.921^b^	0.387 ± 0.008^b^
1:0.4	92.075 ± 2.696^c^	0.963 ± 0.028^c^
1:0.6	79.505 ± 1.134^d^	1.254 ± 0.012^d^
1:0.8	90.776 ± 0.850^d^	1.900 ± 0.017^e^
1:1	94.959 ± 0.700^d^	2.484 ± 0.018^f^

Values are mean (n = 3) ± standard error. Different letter represent significant differences at *p* value <0.05 according to ANOVA and Tukey’s multiple comparison test.

### *In-vitro* release behavior of AREO-CsNe

**Figure 1:**
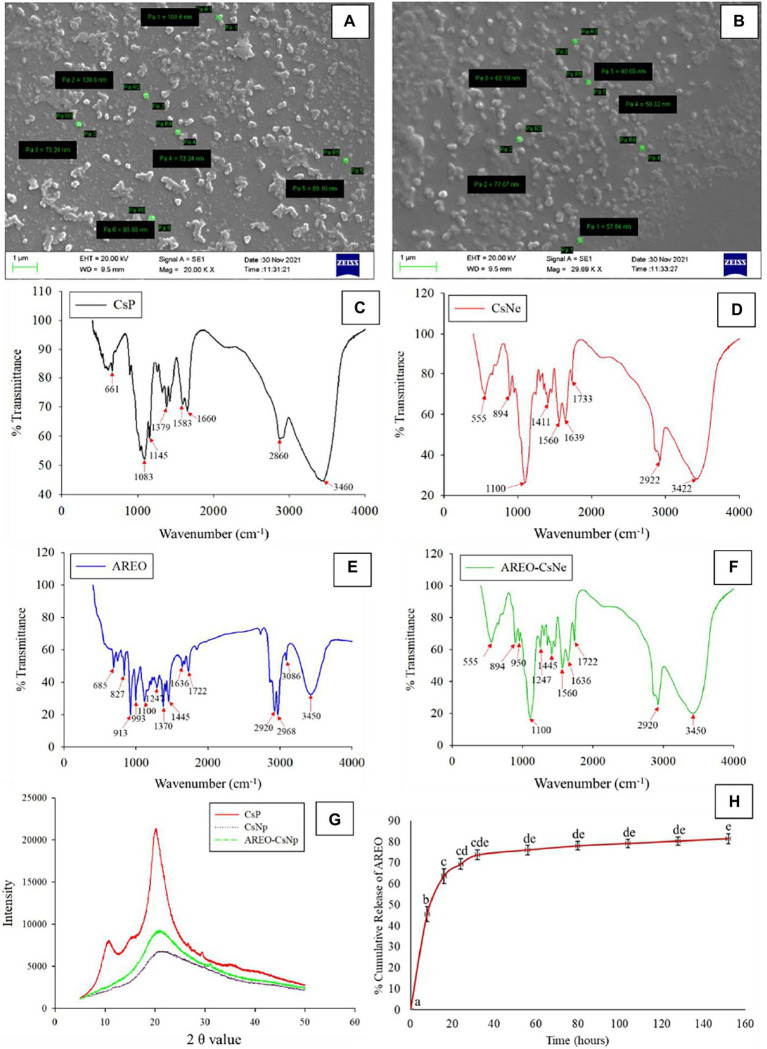
Scanning electron microscopic image of CsNe **(A)** Pa1 = 103.6, Pa2 = 120.8, Pa3 = 73.24, Pa4 = 73.24, Pa5 = 89.10, and Pa6 = 93.80 nm, respectively and AREO-CsNe **(B)** Pa1 = 57.54, Pa2 = 77.07, Pa3 = 63.18, Pa4 = 50.32, and Pa5 = 40.69 nm, respectively, FTIR spectra of CsP, CsNp, AREO, and AREO-CsNe **(C–F)**, X-ray diffraction patterns of CsP, CsNp, and AREO-CsNe **(G)**, and *in-vitro* release profile of AREOX-ray from AREO-CsNe nanoemulsion **(H)**.

*In-vitro* release profile of AREO-CsNe was determined at regular time intervals between 8–152 h (Figure 1H). Release of AREO from AREO-CsNe complex depicted biphasic release with initial burst release (45.55%) within 8 h followed by controlled release after 24, 56, 104, and 152 h, respectively. Similar results with an initial release of 82% encapsulated oregano EO followed by controlled release of remaining EO was reported by Hosseini et al. (2013) during synthesis and characterization of chitosan–TPP nanoparticles loaded with EO. The initial burst release at initial stage might be attributed to the weak interaction between different constituents of AREO and chitosan as well as release of the EO that are adsorbed on the surface of polymer cage (Anitha et al., 2011; Esmaeili and Asgari, 2015). However, the controlled release in later stage could be due to the diffusion of the encapsulated AREO dispersed into the chitosan matrix as the main mechanism (Ji et al., 2019). On the basis of observed results, the AREO-CsNe emulsion showed strong candidature for controlled and sustained delivery of AREO for long term preservation of stored food commodities against fungal infestation and AFB_1_ contamination.

### Physico-chemical characterization of AREO-CsNe

#### Scanning electron microscopy

Particle size of CsNe and AREO-CsNe are presented in Figures 1A,B. Size of CsNe particles ranged between 120.8–73.24 nm, while size of the CsNe particles after incorporation of AREO (AREO-CsNe) was significantly decreased (77.07–40.69 nm). Decrement in size of nanoparticles after encapsulation of AREO might be due to close binding with Cs and S-TPP (Esmaeili and Asgari, 2015). Moreover, the use of Tween-80 as surfactant might be attributed to the reduction in size of nanoemulsion (da Silva Gündel et al., 2018). Aggregation of particles was recorded at some places which has been associated with clustering of one particle with another during freeze drying (Hasheminejad et al., 2019). Our findings are in corroboration with previous investigations of Ziaee et al. (2014) and Dwivedy et al. (2018) demonstrating reduction in size of chitosan nanoparticles after incorporation of *Carum copticum* and *Illicium verum* EOs, respectively. Hence, nanometric size with greater surface to volume ratio of AREO-CsNe may facilitate better inhibitory activity against fungal growth and aflatoxin production.

#### Fourier transform infrared spectroscopy

During FTIR analyses (Figures 1C–F), CsP showed a series of peaks at 3460 cm^−1^ (-OH and-NH stretching), 2,860 cm^−1^ (-CH stretching), 1,660 cm^−1^ (open-chain imino-C=N-stretching), 1,583 cm^−1^ (-C=C-stretching), 1,379 cm^−1^ (-CH stretching), 1,145 cm^−1^ (-C-O-stretching), 1,083 cm^−1^ (primary amine-CN stretching), and 661 cm^−1^ (-CH stretching). For CsNe, peaks 3,460, 2,860, and 1,660 cm^−1^ shifted to 3,422, 2,922, and 1,639 cm^−1^, respectively and new peaks were developed at 1560 cm^−1^ (-NH stretching), 1,411 cm^−1^ (-OH and-CH stretching), 1,100 cm^−1^ (P-O-C stretching), and 894 cm^−1^ (-CH stretching). The P-O stretching indicated ionic interaction between amine groups of chitosan and phosphoric groups of S-TPP. The confirmation on complex formation due to interaction between NH^3+^ groups of chitosan and phosphoric groups of TPP within the nanoparticles was also suggested by Yoksan et al. (2010) during encapsulating ascorbyl palmitate in chitosan nanoparticles. AREO showed a number of peaks at 3450 cm^−1^ (-OH stretching), 3,086 cm^−1^ (-CH stretching), 2,968, 2,920, 1722 cm^−1^ (-CH stretching), 1,636 cm^−1^ (-C=N-and-NH stretching), 1,445, 1,370 cm^−1^ (-CH stretching), 1,247 cm^−1^ (-P-O-stretching), 993, 913 cm^−1^, 827 cm^−1^ (C-O-O-stretching), and 685 cm^−1^ (C-S stretching; Coates, 2000). The presence of numerous peaks in EO revealed the occurrence of different bioactive compounds, and most of the peaks of CsNe and AREO in AREO-CsNe confirmed successful loading of AREO. This behavior of successful loading has been also confirmed by Chaudhari et al. (2022a); Upadhyay et al. (2021), and Das et al. (2022) during encapsulating anethole, *C. odorata* EO, and synergistic mixture of *Pimpinella anismum* and *Coriandrum sativum* essential oil in chitosan nanomatrices. The authors suggested that this behavior might be attributed to the establishment of noncovalent and electrostatic interaction of test compounds and EOs with chitosan-TPP *via* hydrogen bonding, hydrophobic and Van der Waal’s forces.

#### X-ray diffraction study

X-ray diffraction (XRD) patterns of CsP, CsNe, and AREO-CsNe are presented in Figure 1G. CsP showed prominent diffraction peak at 2θ value at 20.3° and a small shoulder peak at 10.9°, indicating high crystalline nature. The tight packaging of constituent molecules of CsP resulted into crystalline and stable nature. For CsNe and AREO-CsNe, broadening of peaks and reduction in peak height depicted the decrement in crystallinity and increase in amorphous nature (Hosseini et al., 2013). The cross linking between NH_3_^+^ of chitosan and PO_4_^3−^ of S-TPP was responsible for broadening of peaks and loss in crystalline nature of CsP (Amalraj et al., 2020). Moreover, the decreased crystalline nature might be due to addition of nanoencapsulants components involved in crystal dissolution at matrices and solvent surfaces along with alteration of diffraction pattern indicating inter and intra molecular interaction between AREO, CsP, and S-TPP (Das et al., 2021c). Study also showed that the width of the peak is related to the degree of polymer crystallinity, and the broadened peak usually results from imperfect crystal. This inferred that the loading of EO resulted in a change in the chitosan-TPP packing structure (Rajkumar et al., 2020). Thus, the alteration of diffraction pattern confirmed successful loading of test EO into chitosan matrix.

### *In-vitro* antifungal and AFB_1_ inhibitory activity of AREO and AREO-CsNe

**Figure 2:**
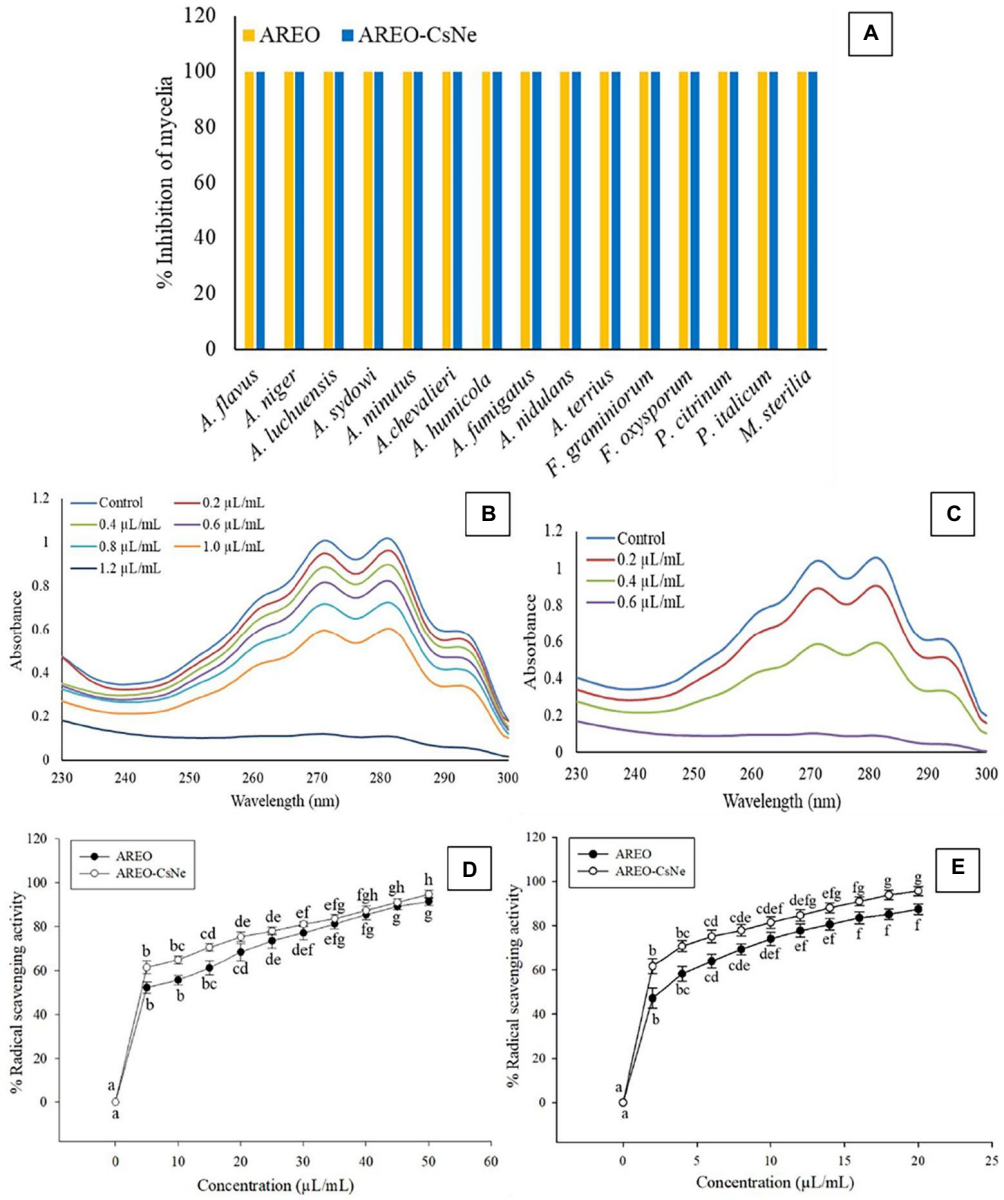
Fungitoxic spectrum of AREO and AREO-CsNe against food contaminating fungi **(A)**, effect of AREO and AREO-CsNe on membrane ergosterol level of AFLHPSi-1 **(B,C)**, antioxidant activity of AREO and AREO-CsNe by DPPH **(D)** and ABTS **(E)** assay.

The MIC and MAIC of AREO was found to be 1.4 and 1.2 μl/ml, respectively, while AREO-CsNe showed enhanced efficacy with MIC and MAIC values 0.8 and 0.6 μl/ml, respectively (Table 3). In the present investigation, TLC method was used to determine AFB_1_ concentration in the conditions of cultured fungi in supplemented SMKY medium. The fungal strains isolated from stored food commodities, when cultured on suitable culture media and incubated in BOD incubator, they produce plenty of AFB_1_ which could be easily quantified by TLC method, which is very common, fast, less expensive, widely used, and requires comparatively lesser amount of organic solvent than High Performance Liquid Chromatography (HPLC). This result of the current study is in good agreement with the earlier study of Beyki et al. (2014) and Kujur et al. (2021), demonstrating enhanced efficacy of *Mentha piperata* and *Eugenia caryophyllata* EOs, respectively against *A. flavus* and aflatoxin biosynthesis after encapsulation in chitosan nanoparticles. The improvement of antifungal and antiaflatoxigenic efficacy of AREO-CsNe over AREO might be attributed to the synergistic action between AREO, and chitosan itself (Amjadi et al., 2019; Chaudhari et al., 2020a). In addition, AREO and AREO-CsNe inhibited the growth of 14 other food borne fungi at their MIC values showing broad spectrum fungitoxic activity (Figure 2A). Increased potency of AREO-CsNe against AFB_1_ secretion may be due to extremely small size of the particles with site-specific delivery of AREO facilitating reduction in fungal sporulation and down regulation of some of the key enzymes involved in AFB_1_ secretion (López-Meneses et al., 2018). The possible reason for AFB_1_ inhibitory effectiveness of AREO and AREO-CsNe was inhibition of spore germination, and impairment in carbohydrate metabolism leading to ultimate production of AFB_1_ in *A. flavus* cells (Das et al., 2021c). Jermnak et al. (2012) reported strong inhibition of norsolorinic acid production as a plausible reason for inhibition of AFB_1_ biosynthesis by essential oil. Hua et al. (2019) and Lasram et al. (2019) pointed out the fact that inhibition of sporulation through velvet gene regulation (veA) and functional disruption of membrane biomolecules as a major factor for restraining of AFB_1_ production by essential oil. Basically, the essential oil and nanoemulsion directly inhibited the production of AFB_1_ by regulating the metabolic steps. Our result agreed with the investigations of Das et al. (2021d) and Chaudhari et al. (2022a), suggesting improvement in antifungal and antiaflatoxigenic activities of *Pimpinella anisum*, and *Pimenta dioca* EOs, respectively.

**Table 3:** Effect of AREO and AREO-CsNe on mycelial weight and aflatoxin B_1_ production by AFLHPSi-1.

Concentration (μL/mL)	AREO				AREO-CsNe			
	Mycelial fresh weight (g)	% Reduction of fresh weight	AFB_1_ (μg/L)	% AFB_1_ reduction	Mycelial fresh weight (g)	% Reduction of fresh weight	AFB_1_ (μg/L)	% AFB_1_ reduction
Control	0.352 ± 0.006^a^	0.000 ± 0.000^a^	4918.532 ± 218.982^a^	0.000 ± 0.000^a^	0.348 ± 0.012^a^	0.000 ± 0.000^a^	4894.678 ± 210.827^a^	0.000 ± 0.000^a^
0.2	0.323 ± 0.008^ab^	8.343 ± 0.809^b^	3196.33 ± 150.18^b^	37.731 ± 5.347^b^	0.282 ± 0.012^b^	19.099 ± 0.916^b^	2857.614 ± 206.795^b^	41.036 ± 6.749^b^
0.4	0.304 ± 0.009^b^	13.507 ± 2.553^b^	1540.917 ± 116.074^c^	68.571 ± 2.544^c^	0.187 ± 0.009^c^	46.372 ± 0.822^c^	524.77 ± 49.806^c^	89.275 ± 0.942^c^
0.6	0.262 ± 0.01^c^	25.617 ± 2.887^c^	963.669 ± 120.405^d^	80.548 ± 1.627^d^	0.022 ± 0.004^d^	93.582 ± 1.415^d^	0.000 ± 0.000^c^	100 ± 0.000^c^
0.8	0.05 ± 0.011^d^	85.531 ± 3.427^d^	634.495 ± 74.519^de^	87.182 ± 0.974^de^	0.000 ± 0.000^d^	100 ± 0.000^e^	–	–
1.0	0.011 ± 0.002^e^	96.881 ± 0.583e	419.816 ± 66.275^ef^	91.550 ± 0.996^ef^	–	–	–	–
1.2	0.004 ± 0.001^e^	98.775 ± 0.409^e^	0.000 ± 0.000^f^	100 ± 0.000^f^	–	–	–	–
1.4	0.000 ± 0.000^e^	100 ± 0.000^e^	–	–	–	–	–	–

AFB_1_ = aflatoxin B_1_; –= Not determined. Values are mean (n = 3) ± standard error; Different letter represent significant differences at *p* value <0.05 according to ANOVA and Tukey’s multiple comparison test.

### Biochemical and molecular docking concerning the mode of action of both AREO and AREO-CsNe

Ergosterol is one of the major cell membrane components in fungal cells responsible for maintaining membrane permeability, and fluidity (Xu et al., 2021; Yang et al., 2021b). Per cent reductions in ergosterol level of AREO fumigated AFLHPSi-1 cells were found to be 7.72, 28.02, 48.88, 68.98, 85.57, 93.47 and 100% at 0.2, 0.4, 0.6, 0.8, 1.0, 1.2, and 1.4 μl/ml, respectively. AREO-CsNe fumigated AFLHPSi-1 cells showed better inhibition as 29.79, 66.40, 93.02, and 100% at 0.2, 0.4, 0.6, and 0.8 μl/ml, respectively (Figures 2B,C; Table 4). Superior efficacy of AREO-CsNe in inhibition of ergosterol biosynthesis might be due to extremely small particle size having better penetration to fungal plasma membrane (Das et al., 2021b). Further, the polycationic nature of chitosan may be responsible for ergosterol inhibition as it can easily binds with the negatively charged components of plasma membrane lipid and abrogate their integrity and functionality. Lin et al. (2003) reported that the fungal cells are susceptible to polyquaternary amines of chitosan, causing disruption of functional and direct insertion of the polymer into the membrane. Other possible actions are that chitosan may enter the fungal cells, interact with their nucleic acid, and alter its functioning *via* conformational changes, chelate with basic proteins, spore elements, and essential nutrients, leading to inhibition of growth (Sajomsang et al., 2012). From the obtained result, it was clear that the test EO and their nanoemulsion would definitely cross the cell wall barrier which is made up of chitin and then inhibit/degrade the ergosterol content, resulting in the disturbance of the plasma membrane integrity and permeability, which subsequently causes leakage of the important cellular contents.

**Table 4:** Effect of AREO and AREO-CsNe on ergosterol reduction of AFLHPSi-1.

Concentration (μL/mL)	AREO		AREO-CsNe	
	Mycelial fresh weight (g)	% Reduction of ergosterol	Mycelial fresh weight (g)	% Reduction of ergosterol
Control	1.012 ± 0.007^a^	0.00 ± 0.00^a^	1.000 ± 0.015^a^	0.000 ± 0.000^a^
0.2	0.958 ± 0.016^ab^	7.729 ± 2.162^a^	0.869 ± 0.002^b^	29.791 ± 2.627^b^
0.4	0.863 ± 0.019^ab^	28.028 ± 2.713^b^	0.646 ± 0.066^c^	66.405 ± 3.322^c^
0.6	0.779 ± 0.034^bc^	48.88 ± 2.363^c^	0.209 ± 0.057^d^	93.029 ± 2.499^d^
0.8	0.670 ± 0.051^c^	68.988 ± 2.572^d^	0.000 ± 0.000^d^	100.000 ± 0.000^d^
1.0	0.428 ± 0.060^d^	85.575 ± 2.587^e^	–	–
1.2	0.194 ± 0.076^e^	93.474 ± 1.862^ef^	–	–
1.4	0.000 ± 0.000^f^	100.000 ± 0.000^f^	–	–

Values are mean (n = 3) ± standard error. Different letter represent significant differences at *p* value <0.05 according to ANOVA and Tukey’s multiple comparison test.

**Figure 3:**
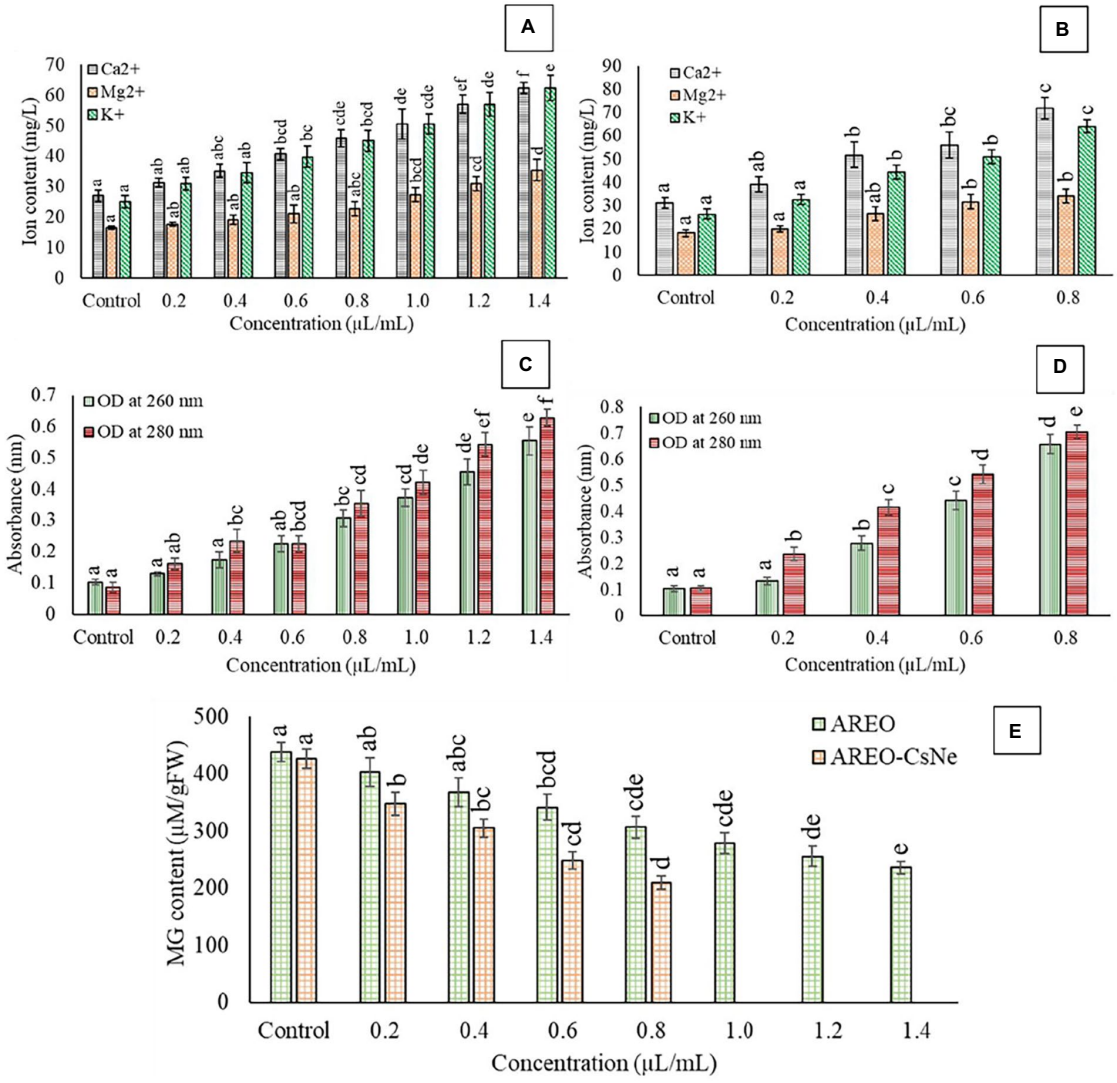
Effect of different concentrations of AREO **(A)** and AREO-CsNe **(B)** on cellular ions leakage **(A,B)**, effect of different concentration of AREO **(C)** and AREO-CsNe **(D)** on 260 and 280 nm absorbing materials leakage, and effect of on cellular methylglyoxal level of AFLHPSi-1 cells **(E)**.

Result of the present investigation depicted dose-dependent increment in loss of Ca^2+^, Mg^2+^, and K^+^ ions as well as 260 and 280 nm absorbing materials from treated AFLHPSi-1 cells (Figures 3A–D); however, nanoemulsion exhibited enhanced efficacy. Cellular ions are involved in maintenance of homeostasis, ATP generation, cellular metabolism, enzymatic secretion, and hyphal growth (Cai et al., 2019). The 260 and 280 nm absorbing materials generally correspond with the concentrations of nucleic acids and proteins, respectively in the cells. The enhanced efficacy of AREO-CsNe over AREO for the leakage of vital cellular ions and UV-absorbing materials might be due to small size, and better mobility through fungal plasma membrane, leading to greater membrane damage and maximum leakage of cellular constituents. Moreover, damaged membrane of fungal cells would not be able to perform the cellular respiration and metabolism which are necessary for growth, development and AFB_1_ production.

MG, a glucose-derived reactive cytotoxic compound synthesized during glycolysis allows production of free radicals and induce secretion of aflatoxin by upregulation of *aflR* and *ver 1* genes (Chen et al., 2004). Therefore, to evaluate antiaflatoxigenic mode of action, herein we had measured MG level in AFLHPSi-1 cells. Control sets showed highest MG level (438.47 and 426.256 μm/g FW), whereas level of MG in AFLHPSi-1 cells fumigated with AREO at 0.2, 0.4, 0.6, 0.8, 1.0, 1.2, and 1.4 μl/ml was found to be 402.45, 367.42, 341.16, 306.49, 278.24, 255.55, and 235.78 μm/g FW, respectively (Figure 3E). AREO-CsNe showed better inhibition of MG biosynthesis in AFLHPSi-1 cells at lower doses than unencapsulated AREO. Our result is in corroboration with previous investigation of Chaudhari et al. (2022b) and Upadhyay et al. (2018), reporting considerable inhibition of MG biosynthesis by nanoencapsulated *Pimenta dioica* and *Cistus ladanifer* EOs, respectively. The antiaflatoxigenic activity might be linked with down regulation of *aflR* and ver-1 genes involved in aflatoxin biosynthesis and the inhibition of MG in fungal cells. Efficient inhibition of MG biosynthesis by AREO-CsNe could be employed for the development of AFB_1_ resistant millet varieties by incorporating sustainable green transgenic approaches in modern agricultural technologies. In the *in-silico* molecular docking (section 3.8.), it has been confirmed that linalool (major component of AREO) potentially interacted with AFB_1_ synthesizing proteins *viz.* Ver-1 and Omt-A, and inhibited AFB_1_ production. In the other way, methylglyoxal production was also alleviated by AREO and AREO-CsNe treatment which was mainly due to –OH group of linalool with effective trapping ability by inhibition of reactive oxygen species production, and electrophilic substitution forming linalool-methylglyoxal adduct formation followed by mitigation of its cellular occurrence. In this way, we have tried to transfer the linalool synthesizing gene (basically linalool synthase; LIS transgene) of *A. rosaeodora* plant into *S. italica* seeds by effective gene transfer technology to develop the AFB_1_ resistant millet varieties, which in one hand has the possibility to combat the methylglyoxal mediated reactive oxygen species production, and in the other hand mitigate the AFB_1_ induced contamination in the storage conditions (Author’s unpublished work). As the LIS gene was isolated from *A. rosaeodora* plant, hence, the green transgenic approaches have been presented with its sustainable application in agricultural technologies.

### *In-silico* molecular docking of linalool with Ver-1 and Omt-A proteins

**Figure 4:**
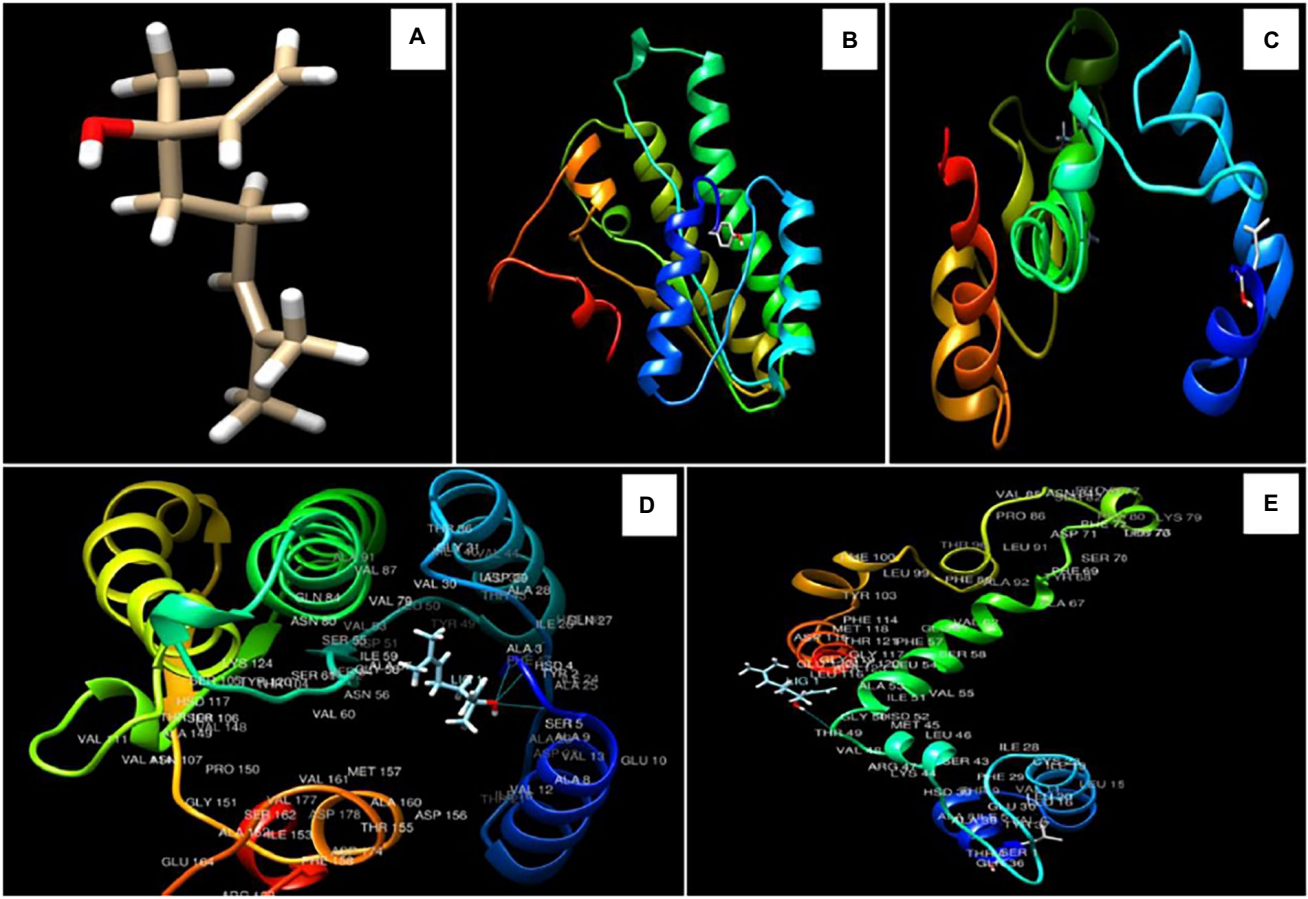
3D structure of linalool **(A)**, Ver-1 and Omt-A protein structures **(B,C)**, interaction of linalool with ALA 3, HSD 4, SER 5, and with THR 49 amino acids in Ver-1 and Omt-A proteins **(D,E)**.

For determination of the molecular target site of action, two different regulatory proteins *viz.* Ver-1 (versicolorin dehydrogenase) and Omt A (sterigmatocystin o-methyltransferase) were selected on the basis of their crucial role in conversion of versicolorin to sterigmatocystin and sterigmatocystin to dihydro-o-methylsterigamatocystin in AFB_1_ biosynthesis, respectively. 3D structure of linalool was downloaded from PUBCHEM data base (Figure 4A), while Ver-1 and Omt-A protein structures were obtained from phyre 2 online server (Figures 4B,C). In the present piece of work, linalool was found to be maximally interacted with ALA 3, HSD 4, SER 5, and THR 49 amino acids in Ver-1 and Omt-A proteins (Figures 4D,E). During molecular interaction, more negative binding energies (−6.140 and-5.766 Kcal/mol) were recorded and bond lengths were found in the range of 1.870–2.439 Å, suggesting efficient interaction of linalool with receptor proteins (Table 5). Our result is in agreement with the previous finding of Murugan et al. (2019) for inhibition of AFB_1_ biosynthesis by molecular interaction of 3,7,11,15-tetramethylhexadec-2-en-1-ol, -Phenylquinazoline-4-Carboximidamide, and methyl palmitate with Ver-1 protein and Chaudhari et al. (2022b) by molecular interaction of major compounds α-pinine, bornyl acetate, and camphor with the target protein Nor-1 primarily catalyze an important step in AFB_1_ biosynthesis. Conclusively, strong interaction of linalool with Ver-1 and Omt A proteins facilitate functional changes which led to inhibition of AFB_1_ biosynthesis. This *in-silico* molecular docking forms a base or podium for further determination of wet lab molecular mechanism of action. However, for further practical application of AREO and its nanoemulsion as green preservative to combat the AFB_1_ contamination in food commodities, investigation for the effect of AREO and its components on Ver-1 and Omt-A genes expression by real time PCR should be worked out.

**Table 5:** Binding energy and bond length of linalool (major component of AREO) with Ver-1 and Omt A protein.

Major component of AREO	Receptor protein	H-bonding amino acid	Binding energy (Kcal/mol)	Bond length (Å)
Linalool	Ver-1	ALA-3	−6.140	2.306
		HSD-4		2.439
		SER-5		2.271
	Omt A	THR-49	−5.766	1.87

### Antioxidant activity of AREO and AREO-CsNe

Since, ROS production and lipid peroxidation leading to deterioration of stored food commodities, the antioxidant activity of AREO and AREO-CsNe was assessed through DPPH^•+^ and ABTS^•+^ assay and expressed in terms of IC_50_ (50% scavenging of free radicals). The IC_50_ value of AREO was found to be 4.468 μl/ml through DPPH^•+^ and 2.370 μl/ml through ABTS^•+^ assay, whereas AREO-CsNe showed enhanced antioxidant activities with IC_50_ values 3.792 μl/ml and 1.706 μl/ml through DPPH^•+^ and ABTS^•+^ assay, respectively (Figures 2D,E). Better antioxidant activity of AREO-CsNe over AREO might be attributed to the better solubility in aqueous solution leading to controlled delivery of active components with better scavenging potentialities of free radicals (Lou et al., 2017; Chaudhari et al., 2020a). Similar reports on enhancement in antioxidant activities after encapsulation of *clove* EO and *Petroselinum crispum* EO into chitosan nanomatrix has been reported by Hadidi et al. (2020) and Deepika et al. (2021), respectively. Chitosan possesses very less antioxidant activity due to intramolecular hydrogen bonding within its polymeric chain which utilised hydrogen atoms from hydroxyl and amine moiety causing reduction in free radical neutralising capacity (Negm et al., 2020). In current scenario, demand of natural antioxidants is increasing rapidly because of negative impacts of synthetic food preservatives (Prakash et al., 2018; Singh et al., 2021). Hence, there is need to develop better and effective natural antioxidants like AREO and AREO-CsNe having better antifungal and antiaflatoxigenic activities with potential application in preservation of stored food commodities.

### *In-situ* antifungal and antiaflatoxigenic efficacy of AREO and AREO-CsNe in *Setaria italica* seeds

Although AREO and AREO-CsNe showed prominent *in-vitro* antifungal and AFB_1_ inhibitory activities with possible mechanism of action, these results are not enough for its potential application in real food systems. Therefore, the *in-situ* efficacy of AREO and AREO-CsNe was assessed in *S. italica* seeds as a model food system and seed samples were fumigated at MIC and 2 MIC doses for 1 year of storage period. During *in-situ* study involving millet food system some of the essential oil content might be absorbed by food commodity itself, hence, 2 MIC concentrations of AREO and AREO-CsNe have been taken into consideration in the present investigation. The present investigation represented 78.69 and 86.32% protection against fungal infestation in stored *S. italica* seeds at MIC and 2 MIC doses of AREO. However, AREO-CsNe at MIC and 2 MIC doses completely inhibited fungal growth in stored *S. italica* seed samples (Table 6). For detection of AFB_1_ in stored *S. italica* seeds, HPLC method was used because AFB_1_ was present in quite small quantity that cannot be quantified precisely by TLC. The maximum content of AFB_1_ as recorded by HPLC in control seed samples was 10.136 μg/kg, whereas the AFB_1_ content in *S. italica* seeds fumigated with MIC dose of AREO was found to be 1.476 μg/kg and complete inhibition of AFB_1_ content was recorded at 2 MIC dose. In case of AREO-CsNe fumigated sets (MIC and 2 MIC doses), complete (100%) inhibition of AFB_1_ production was recorded (Table 6). In the present investigation, higher dose of AREO was required for complete inhibition of AFB_1_ in *S. italica* seeds which may be due to degradation of some of the volatile components of EO and absorption/adsorption by the stored food commodity itself. Superior AFB_1_ inhibitory activity of AREO-CsNe in stored food system might be due to nano-range size of particles and controlled release over a longer period of time (Das et al., 2019; Hossain et al., 2019). The results obtained in present investigation suggested the practical application of nanoencapsulated AREO as next generation green preservative against AFB_1_ mediated biodeterioration of stored millets.

**Table 6:** Inhibition of fungal growth and AFB1 production in *S. italica* seeds by AREO and AREO-CsNe fumigation.

Treatment sets	Number of fungal colonies	% Protection	AFB_1_ content (μg/kg)	% Protection
Control	56 ± 2.01^a^	0.00 ± 0.00^a^	10.136 ± 1.14^a^	0.00 ± 0.00^a^
AREO (MIC dose)	12 ± 1.67^b^	78.69 ± 3.64^b^	1.476 ± 0.04^b^	85.43 ± 3.16^b^
AREO (2 MIC dose)	8.0 ± 1.31^c^	86.32 ± 2.07^c^	0.00 ± 0.00^c^	100 ± 0.00^c^
AREO-CsNe (MIC dose)	0.00 ± 0.00^d^	100 ± 0.00^d^	0.00 ± 0.00^c^	100 ± 0.00^c^
AREO-CsNe (2 MIC dose)	0.00 ± 0.00^d^	100 ± 0.00^d^	0.00 ± 0.00^c^	100 ± 0.00^c^

Values are mean (n = 3) ± standard error. Different letter represent significant differences at *p* value <0.05 according to ANOVA and Tukey’s multiple comparison test.

### Effect of AREO and AREO-CsNe on inhibition of lipid peroxidation

**Figure 5:**
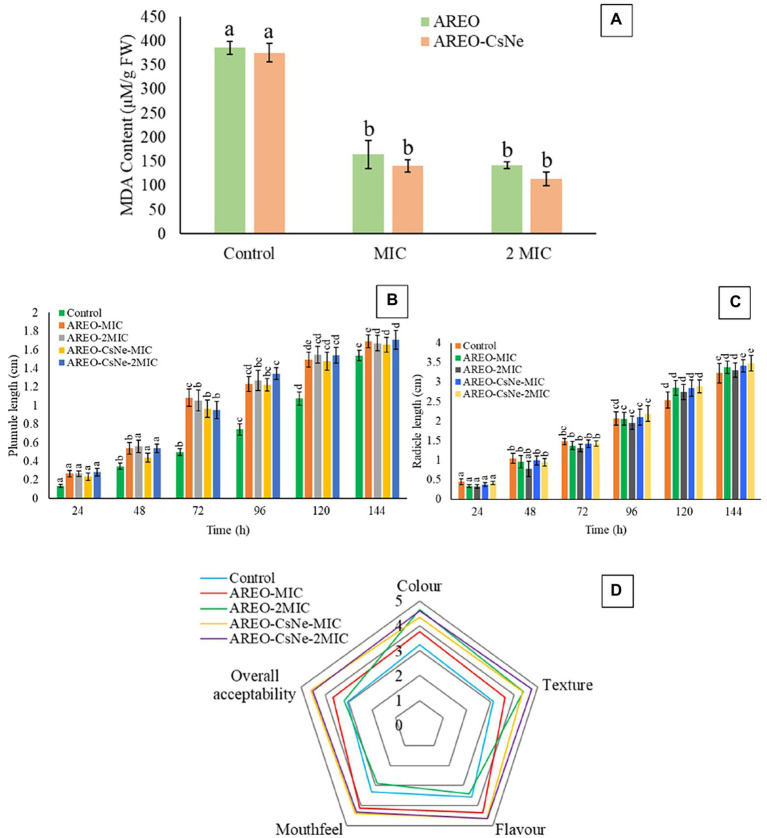
Effect of AREO and AREO-CsNe on of lipid peroxidation **(A)**, phytotoxicity assay **(B)** 907, **(C)**, and sensory evaluation **(D)** of *S. italica* seed samples treated with AREO and AREO-CsNe at MIC and 2MIC dose.

Lipid peroxidation is one of the major biodeteriorating processes responsible for depletion of nutrient content, off-flavour, and off-taste of stored food commodities. Malondialdehyde (MDA) is produced as result of peroxidation of polyunsaturated fatty acids in stored food system. In the present investigation, thiobarbituric acid (TBA) assay was used for quantification of MDA content (Xu et al., 2009). The MDA content of control sets were found to be 385.806 and 375.268 μm/gFW, while in case of *S. italica* seeds fumigated at MIC and 2 MIC doses of AREO, the MDA content reduced to 164.515 and 142.795 μm/gFW, respectively. AREO-CsNe depicted better inhibition of MDA content at MIC (140.64 μm/g fresh weight) and 2 MIC doses (114.193 μm/g fresh weight; Figure 5A). The enhanced efficacy of nanoencapsulated AREO in MDA reduction might be associated with controlled release of AREO from chitosan nanomatrix causing maximum scavenging of biodeteriorating free radicals.

### Phytotoxicity assessment of AREO and AREO-CsNe

The AREO and AREO-CsNe fumigated *S. italica* seeds showed non-phytotoxic effect as observed by emergence of plumule and radicles within 24–120 h (Figures 5B,C). Non-phytotoxic nature of AREO and AREO-CsNe on seed germination strengthens its candidature as natural food preservative and fumigated seeds may be used further for sowing purposes in next growing season and other intended agricultural practices.

### Assessment of safety profile of AREO and AREO-CsNe

The LD_50_ values of AREO and AREO-CsNe were found to be 8142.742 μl/kg and 9538.742 μl/kg body weight, respectively. The LD_50_ values were found higher than commonly used botanical preservatives such as azadirachtin (~5,000 mg/kg), pyrethrum (350–500 mg/kg), and recommended cut-off (>5,000 mg/kg) of Organisation for Economic Co-operation and Development (OECD) guidelines suggesting practical recommendation in food industries (Coats, 1994; Isman, 2006). The obtained LD_50_ value of AREO-CsNe was found far greater than that of some frequently reported commercial compounds such as pyrethrum, bavistin, and formic acid as well as some EOs *viz.*, *Artemisia dracunculus* and *Melaleuca cajuputi* EOs (Prakash et al., 2012; Chaudhari et al., 2022b). This result confirmed mammalian non-toxicity of the nanoemulsion and, hence, can be considered for application as food preservative of stored food products.

### Sensory properties of *Setaria italica* seeds fumigated with AREO and AREO-CsNe

Effect of AREO and AREO-CsNe on sensory properties of *S. italica* seeds for 1 year of storage was assessed by 5-point hedonic scale as presented in Figure 5D. At the MIC dose of AREO, there was improvement in scores for colour, texture, odour, mouth feel, and overall acceptability, while there was decrement in scores for mouth feel and flavour recorded in samples fumigated with 2 MIC doses. This decrement in scores for mouth feel and flavour might be due to absorption of EO by stored commodities at higher doses. However, AREO-CsNe fumigated seeds showed improvement in scores without any negative impacts on colour, texture, odour, mouth feel, and overall acceptability at both MIC and 2 MIC doses. This result is consistent with the study of Chaudhari et al. (2020b) and Tiwari et al. (2022), who suggested improvement in sensory parameters of maize and black cumin seeds after fumigation with *Origanum majorana* and *Cinnamomum glaucescens* EOs loaded chitosan nanoemulsion. The acceptable result in case of AREO-CsNe has been associated with controlled release of EO from chitosan nanomatrix preventing the absorption of EO by stored food commodities. Thus, the maintenance of sensory properties of stored *S. italica* seeds after fumigation with AREO-CsNe strengthens its utilization as green preservative in food and agricultural industries.

## Conclusion

The results of the present investigation showed that AREO-CsNe exhibited enhanced antifungal, antiaflatoxigenic and antioxidant activities under both *in-vitro* and *in-situ* conditions. The antifungal and antiaflatoxigenic mode of action was linked to the disruption of plasma membrane integrity and MG inhibition, respectively. Further, the AREO-CsNe showed remarkable efficacy in protection of stored millets for 1 year against fungal infestation, AFB_1_ contamination, and lipid peroxidation and presented satisfactory safety profile and acceptable sensory properties. Overall, AREO-CsNe can be recommended as safe and eco-friendly plant-based preservative to improve the shelf-life of stored millets and other agricultural commodities.

## Data availability statement

The original contributions presented in the study are included in the article/Supplementary material, further inquiries can be directed to the corresponding author.

## Ethics statement

The animal study was reviewed and approved by Central Animal Ethical Committee, Institute of Medical Sciences, Banaras Hindu University.

## Author contributions

BS: conceptualization, methodology, writing original draft, and funding acquisition. AC, SD, and VS: review and editing. ST and AM: data curation. ND: supervision and writing—review and editing. All authors contributed to the article and approved the submitted version.

## Funding

Funding was provided in the form of research fellowship by Council of Scientific and Industrial Research (CSIR) [grant no. 09/013(0920)/2019-EMR-I].

## Conflict of interest

The authors declare that the research was conducted in the absence of any commercial or financial relationships that could be construed as a potential conflict of interest

## Publisher’s note

All claims expressed in this article are solely those of the authors and do not necessarily represent those of their affiliated organizations, or those of the publisher, the editors and the reviewers. Any product that may be evaluated in this article, or claim that may be made by its manufacturer, is not guaranteed or endorsed by the publisher.

## References

[ref1] Adams R. P. (2007). Identification of Essential oil Components by gas Chromatography/mass Spectrometry (Vol. 456). Carol Stream, IL: Allured publishing corporation.

[ref2] Ajiboye A. A. Dedeke O. A. Adeyemo F. C. (2017). Investigation on antioxidants, free radical scavenger and lipid peroxidation activities of whole grains finger millet (*Eleusine coracana* L.). Int. J. Plant Biol. 8, 6684. doi: 10.4081/pb.2017.6684

[ref3] Alehosseini E. Jafari S. M. Tabarestani H. S. (2021). Production of d-limonene-loaded Pickering emulsions stabilized by chitosan nanoparticles. Food Chem. 354:129591. doi: 10.1016/j.foodchem.2021.129591, PMID: 33756315

[ref4] Amalraj A. Haponiuk J. T. Thomas S. Gopi S. (2020). Preparation, characterization and antimicrobial activity of polyvinyl alcohol/gum arabic/chitosan composite films incorporated with black pepper essential oil and ginger essential oil. Int. J. Biol. Macromol. 151, 366–375. doi: 10.1016/j.ijbiomac.2020.02.176, PMID: 32084477

[ref5] Amjadi S. Emaminia S. Nazari M. Davudian S. H. Roufegarinejad L. Hamishehkar H. (2019). Application of reinforced ZnO nanoparticle-incorporated gelatin bionanocomposite film with chitosan nanofiber for packaging of chicken fillet and cheese as food models. Food Bioprocess Technol. 12, 1205–1219. doi: 10.1007/s11947-019-02286-y

[ref6] Anitha A. Deepagan V. G. Rani V. D. Menon D. Nair S. V. Jayakumar R. (2011). Preparation, characterization, in vitro drug release and biological studies of curcumin loaded dextran sulphate–chitosan nanoparticles. Carbohydr. Polym. 84, 1158–1164. doi: 10.1016/j.carbpol.2011.01.005

[ref7] Bagheri R. Ariaii P. Motamedzadegan A. (2021). Characterization, antioxidant and antibacterial activities of chitosan nanoparticles loaded with nettle essential oil. J. Food Meas Charact. 15, 1395–1402. doi: 10.1007/s11694-020-00738-0

[ref8] Bandyopadhyay T. Jaiswal V. Prasad M. (2017a). “Nutrition potential of foxtail millet in comparison to other millets and major cereals,” in The Foxtail Millet Genome. ed. M. Prasad (Cham: Springer), 123–135.

[ref9] Bandyopadhyay T. Muthamilarasan M. Prasad M. (2017b). Millets for next generation climate-smart agriculture. Front. Plant Sci. 8:1266. doi: 10.3389/fpls.2017.01266, PMID: 28769966 PMC5513978

[ref10] Beyki M. Zhaveh S. Khalili S. T. Rahmani-Cherati T. Abollahi A. Bayat M.. (2014). Encapsulation of *Mentha piperita* essential oils in chitosan–cinnamic acid nanogel with enhanced antimicrobial activity against *Aspergillus flavus*. Ind. Crop. Prod. 54, 310–319. doi: 10.1016/j.indcrop.2014.01.033

[ref11] Cai R. Hu M. Zhang Y. Niu C. Yue T. Yuan Y.. (2019). Antifungal activity and mechanism of citral, limonene and eugenol against *Zygosaccharomyces rouxii*. LWT 106, 50–56. doi: 10.1016/j.lwt.2019.02.059

[ref700] Chaudhari A. K. Dwivedy A. K. Singh V. K. Das S. Singh A. Dubey N. K. (2019). Essential oils and their bioactive compounds as green preservatives against fungal and mycotoxin contamination of food commodities with special reference to their nanoencapsulation. Environ. Sci. Pollut. Res. 26, 25414–25431. doi: 10.1007/s11356-019-05932-2 31313235

[ref12] Chaudhari A. K. Singh V. K. Das S. Dubey N. K. (2022a). Fabrication, characterization, and bioactivity assessment of chitosan nanoemulsion containing allspice essential oil to mitigate *Aspergillus flavus* contamination and aflatoxin B_1_ production in maize. Food Chem. 372:131221. doi: 10.1016/j.foodchem.2021.131221, PMID: 34649029

[ref13] Chaudhari A. K. Singh V. K. Das S. Kujur A. Dubey N. K. (2022b). Unveiling the cellular and molecular mode of action of *Melaleuca cajuputi* Powell. Essential oil against aflatoxigenic strains of *Aspergillus flavus* isolated from stored maize samples. Food Control 138:109000. doi: 10.1016/j.foodcont.2022.109000

[ref14] Chaudhari A. K. Singh V. K. Das S. Prasad J. Dwivedy A. K. Dubey N. K. (2020b). Improvement of *in vitro* and *in situ* antifungal, AFB_1_ inhibitory and antioxidant activity of *Origanum majorana* L. essential oil through nanoemulsion and recommending as novel food preservative. Food Chem. Toxicol. 143:111536. doi: 10.1016/j.fct.2020.111536, PMID: 32640350

[ref15] Chaudhari A. K. Singh V. K. Das S. Singh B. K. Dubey N. K. (2020a). Antimicrobial, aflatoxin B_1_ inhibitory and lipid oxidation suppressing potential of anethole-based chitosan nanoemulsion as novel preservative for protection of stored maize. Food Bioprocess Technol. 13, 1462–1477. doi: 10.1007/s11947-020-02479-w

[ref16] Chaudhari A. K. Singh V. K. Dwivedy A. K. Das S. Upadhyay N. Singh A.. (2020c). Chemically characterised *Pimenta dioica* (L.) Merr. Essential oil as a novel plant based antimicrobial against fungal and aflatoxin B_1_ contamination of stored maize and its possible mode of action. Nat. Prod. Res. 34, 745–749. doi: 10.1080/14786419.2018.1499634, PMID: 30421964

[ref17] Chen Z. Y. Brown R. L. Damann K. E. Cleveland T. E. (2004). Identification of a maize kernel stress-related protein and its effect on aflatoxin accumulation. Phytopathology 94, 938–945. doi: 10.1094/PHYTO.2004.94.9.938, PMID: 18943070

[ref18] Chibuzor-Onyema I. E. Ezeokoli O. T. Sulyok M. Notununu I. Petchkongkaew A. Elliott C. T.. (2021). Metataxonomic analysis of bacterial communities and mycotoxin reduction during processing of three millet varieties into Ogi, a fermented cereal beverage. Food Res. Int. 143:110241. doi: 10.1016/j.foodres.2021.110241, PMID: 33992353

[ref19] Coates J., (2000). Interpretation of infrared spectra, a practical approach.

[ref20] Coats J. R. (1994). Risks from natural versus synthetic insecticides. Annu. Rev. Entomol. 39, 489–515. doi: 10.1146/annurev.en.39.010194.002421, PMID: 8135501

[ref21] Contigiani E. V. Jaramillo-Sánchez G. Castro M. A. Gómez P. L. Alzamora S. M. (2018). Postharvest quality of strawberry fruit (*Fragaria x Ananassa* Duch cv. Albion) as affected by ozone washing: fungal spoilage, mechanical properties, and structure. Food Bioprocess Technol. 11, 1639–1650. doi: 10.1007/s11947-018-2127-0

[ref23] da Silva Gündel S. de Souza M. E. Quatrin P. M. Klein B. Wagner R. Gündel A.. (2018). Nanoemulsions containing *Cymbopogon flexuosus* essential oil: development, characterization, stability study and evaluation of antimicrobial and antibiofilm activities. Microb. Pathog. 118, 268–276. doi: 10.1016/j.micpath.2018.03.043, PMID: 29581028

[ref24] Das S. Singh V. K. Chaudhari A. K. Dwivedy A. K. Dubey N. K. (2022). Co-encapsulation of *Pimpinella anisum* and *Coriandrum sativum* essential oils based synergistic formulation through binary mixture: Physico-chemical characterization, appraisal of antifungal mechanism of action, and application as natural food preservative. Pestic. Biochem. Physiol. 184, 105066. doi: 10.1016/j.pestbp.2022.105066, PMID: 35715028

[ref25] Das S. Singh V. K. Dwivedy A. K. Chaudhari A. K. Dubey N. K. (2021a). Insecticidal and fungicidal efficacy of essential oils and nanoencapsulation approaches for the development of next generation ecofriendly green preservatives for management of stored food commodities: an overview. Int. J. Pest Manag., 1–32. doi: 10.1080/09670874.2021.1969473

[ref26] Das S. Singh V. K. Dwivedy A. K. Chaudhari A. K. Dubey N. K. (2021b). Eugenol loaded chitosan nanoemulsion for food protection and inhibition of Aflatoxin B_1_ synthesizing genes based on molecular docking. Carbohydr. Polym. 255:117339. doi: 10.1016/j.carbpol.2020.117339, PMID: 33436182

[ref27] Das S. Singh V. K. Dwivedy A. K. Chaudhari A. K. Dubey N. K. (2021c). *Anethum graveolens* essential oil encapsulation in chitosan nanomatrix: investigations on in vitro release behavior, organoleptic attributes, and efficacy as potential delivery vehicles against biodeterioration of rice (*Oryza sativa* L.). Food Bioprocess Technol. 14, 831–853. doi: 10.1007/s11947-021-02589-z

[ref28] Das S. Singh V. K. Dwivedy A. K. Chaudhari A. K. Dubey N. K. (2021d). Nanostructured *Pimpinella anisum* essential oil as novel green food preservative against fungal infestation, aflatoxin B_1_ contamination and deterioration of nutritional qualities. Food Chem. 344:128574. doi: 10.1016/j.foodchem.2020.128574, PMID: 33218855

[ref29] Das S. Singh V. K. Dwivedy A. K. Chaudhari A. K. Upadhyay N. Singh P.. (2019). Encapsulation in chitosan-based nanomatrix as an efficient green technology to boost the antimicrobial, antioxidant and in situ efficacy of *Coriandrum sativum* essential oil. Int. J. Biol. Macromol. 133, 294–305. doi: 10.1016/j.ijbiomac.2019.04.070, PMID: 30986458

[ref30] Das S. Singh V. K. Dwivedy A. K. Chaudhari A. K. Upadhyay N. Singh A.. (2020). Fabrication, characterization and practical efficacy of *Myristica fragrans* essential oil nanoemulsion delivery system against postharvest biodeterioration. Ecotoxicol. Environ. Saf. 189:110000. doi: 10.1016/j.ecoenv.2019.110000, PMID: 31787384

[ref31] Deepika C. A. K. Singh A. Das S. Dubey N. K. (2021). Nanoencapsulated *Petroselinum crispum* essential oil: characterization and practical efficacy against fungal and aflatoxin contamination of stored chia seeds. Food Biosci. 42:101117. doi: 10.1016/j.fbio.2021.101117

[ref33] Duodu K. G. Dowell F. E. (2019). “Sorghum and millets: quality management systems,” in Sorghum and Millets. eds. J. R. N. Taylor and K. G. Duodu (United Kingdom: AACC International Press), 421–442.

[ref34] Dwivedy A. K. Singh V. K. Prakash B. Dubey N. K. (2018). Nanoencapsulated *Illicium verum* Hook. f. Essential oil as an effective novel plant-based preservative against aflatoxin B_1_ production and free radical generation. Food Chem. Toxicol. 111, 102–113. doi: 10.1016/j.fct.2017.11.007, PMID: 29126800

[ref35] Esmaeili A. Asgari A. (2015). In vitro release and biological activities of *Carum copticum* essential oil (CEO) loaded chitosan nanoparticles. Int. J. Biol. Macromol. 81, 283–290. doi: 10.1016/j.ijbiomac.2015.08.010, PMID: 26257380

[ref36] Finney D. J., (1971). Probit analysis, Cambridge University Press. Cambridge, UK.

[ref37] Froiio F. Mosaddik A. Morshed M. T. Paolino D. Fessi H. Elaissari A. (2019). Edible polymers for essential oils encapsulation: application in food preservation. Ind. Eng. Chem. Res. 58, 20932–20945. doi: 10.1021/acs.iecr.9b02418

[ref38] Gilman J. C. Joseph C. (1998). A Manual of Soil Fungi, Delhi: Daya Books.

[ref39] Hadidi M. Pouramin S. Adinepour F. Haghani S. Jafari S. M. (2020). Chitosan nanoparticles loaded with clove essential oil: characterization, antioxidant and antibacterial activities. Carbohydr. Polym. 236:116075. doi: 10.1016/j.carbpol.2020.116075, PMID: 32172888

[ref40] Hasheminejad N. Khodaiyan F. Safari M. (2019). Improving the antifungal activity of clove essential oil encapsulated by chitosan nanoparticles. Food Chem. 275, 113–122. doi: 10.1016/j.foodchem.2018.09.085, PMID: 30724177

[ref41] Hossain F. Follett P. Vu K. D. Salmieri S. Fraschini C. Jamshidian M.. (2019). Antifungal activity of combined treatments of active methylcellulose-based films containing encapsulated nanoemulsion of essential oils and γ–irradiation: in vitro and in situ evaluations. Cellulose 26, 1335–1354. doi: 10.1007/s10570-018-2135-2

[ref42] Hosseini S. F. Zandi M. Rezaei M. Farahmandghavi F. (2013). Two-step method for encapsulation of oregano essential oil in chitosan nanoparticles: preparation, characterization and in vitro release study. Carbohydr. Polym. 95, 50–56. doi: 10.1016/j.carbpol.2013.02.031, PMID: 23618238

[ref43] Hu Q. Luo Y. (2021). Chitosan-based nanocarriers for encapsulation and delivery of curcumin: A review. Int. J. Biol. Macromol. 179, 125–135. doi: 10.1016/j.ijbiomac.2021.02.216, PMID: 33667554

[ref44] Hua S. S. T. Sarreal S. B. L. Chang P. K. Yu J. (2019). Transcriptional regulation of aflatoxin biosynthesis and conidiation in *Aspergillus flavus* by *Wickerhamomyces anomalus* WRL-076 for reduction of aflatoxin contamination. Toxins 11, 81. doi: 10.3390/toxins11020081, PMID: 30717146 PMC6410245

[ref45] Isman M. B. (2006). Botanical insecticides, deterrents, and repellents in modern agriculture and an increasingly regulated world. Annu. Rev. Entomol. 51, 45–66. doi: 10.1146/annurev.ento.51.110104.151146, PMID: 16332203

[ref46] Isman M. B. (2020). Botanical insecticides in the twenty-first century—fulfilling their promise? Annu. Rev. Entomol. 65, 233–249. doi: 10.1146/annurev-ento-011019-025010, PMID: 31594414

[ref47] Jaiswal V. Bandyopadhyay T. Gahlaut V. Gupta S. Dhaka A. Ramchiary N.. (2019). Genome-wide association study (GWAS) delineates genomic loci for ten nutritional elements in foxtail millet (*Setaria italica* L.). J. Cereal Sci. 85, 48–55. doi: 10.1016/j.jcs.2018.11.006

[ref48] Jermnak U. Yoshinari T. Sugiyama Y. Tsuyuki R. Nagasawa H. Sakuda S. (2012). Isolation of methyl syringate as a specific aflatoxin production inhibitor from the essential oil of *Betula alba* and aflatoxin production inhibitory activities of its related compounds. Int. J. Food Microbiol. 153, 339–344. doi: 10.1016/j.ijfoodmicro.2011.11.023, PMID: 22177852

[ref49] Ji M. Sun X. Guo X. Zhu W. Wu J. Chen L.. (2019). Green synthesis, characterization and *in vitro* release of cinnamaldehyde/sodium alginate/chitosan nanoparticles. Food Hydrocoll. 90, 515–522. doi: 10.1016/j.foodhyd.2018.12.027

[ref50] Ju J. Chen X. Xie Y. Yu H. Guo Y. Cheng Y.. (2019). Application of essential oil as a sustained release preparation in food packaging. Trends Food Sci. Technol. 92, 22–32. doi: 10.1016/j.tifs.2019.08.005

[ref52] Kujur A. Kumar A. Prakash B. (2021). Elucidation of antifungal and aflatoxin B1 inhibitory mode of action of *Eugenia caryophyllata* L. essential oil loaded chitosan nanomatrix against *Aspergillus flavus*. Pestic. Biochem. Physiol. 172:104755. doi: 10.1016/j.pestbp.2020.104755, PMID: 33518049

[ref53] Lasram S. Zemni H. Hamdi Z. Chenenaoui S. Houissa H. Tounsi M. S.. (2019). Antifungal and antiaflatoxinogenic activities of *Carum carvi* L., *Coriandrum sativum* L. seed essential oils and their major terpene component against *Aspergillus flavus*. Ind. Crop. Prod. 134, 11–18. doi: 10.1016/j.indcrop.2019.03.037

[ref54] Lin J. Qiu S. Lewis K. Klibanov A. M. (2003). Mechanism of bactericidal and fungicidal activities of textiles covalently modified with alkylated polyethylenimine. Biotechnol. Bioeng. 83, 168–172. doi: 10.1002/bit.10651, PMID: 12768622

[ref55] López-Meneses A. K. Plascencia-Jatomea M. Lizardi-Mendoza J. Fernández-Quiroz D. Rodríguez-Félix F. Mouriño-Pérez R. R.. (2018). *Schinus molle* L. essential oil-loaded chitosan nanoparticles: preparation, characterization, antifungal and anti-aflatoxigenic properties. LWT 96, 597–603. doi: 10.1016/j.lwt.2018.06.013

[ref56] Lou Z. Chen J. Yu F. Wang H. Kou X. Ma C.. (2017). The antioxidant, antibacterial, antibiofilm activity of essential oil from *Citrus medica* L. var. sarcodactylis and its nanoemulsion. LWT 80, 371–377. doi: 10.1016/j.lwt.2017.02.037

[ref57] Maia J. G. S. Mourão R. H. V. (2016). “Amazon rosewood (*Aniba rosaeodora* Ducke) oils,” in Essential Oils in Food Preservation, Flavor and Safety (United States: Academic Press), 193–201.

[ref701] Murugan C. Sharma V. Murugan R. K. Malaimegu G. Sundaramurthy A. (2019). Two-dimensional cancer theranostic nanomaterials: Synthesis, surface functionalization and applications in photothermal therapy. J. Control. Release. 299, 1–20. doi: 10.1016/j.jconrel.2019.02.015, PMID: 30771414

[ref58] Nadeem F. Ahmad Z. Wang R. Han J. Shen Q. Chang F.. (2018). Foxtail millet [*Setaria italica* (L.) Beauv.] grown under low nitrogen shows a smaller root system, enhanced biomass accumulation, and nitrate transporter expression. Front. Plant Sci. 9:205. doi: 10.3389/fpls.2018.00205, PMID: 29520286 PMC5826958

[ref59] Nattudurai G. Baskar K. Paulraj M. G. Islam V. I. H. Ignacimuthu S. Duraipandiyan V. (2017). Toxic effect of *Atalantia monophylla* essential oil on *Callosobruchus maculatus* and *Sitophilus oryzae*. Environ. Sci. Pollut. Res. 24, 1619–1629. doi: 10.1007/s11356-016-7857-9, PMID: 27796969

[ref60] Negm N. A. Kana M. T. H. A. Abubshait S. A. Betiha M. A. (2020). Effectuality of chitosan biopolymer and its derivatives during antioxidant applications. Int. J. Biol. Macromol. 164, 1342–1369. doi: 10.1016/j.ijbiomac.2020.07.197, PMID: 32726651

[ref61] OuYang Q. Okwong R. O. Chen Y. Tao N. (2020). Synergistic activity of cinnamaldehyde and citronellal against green mold in citrus fruit. Postharvest Biol. Technol. 162:111095. doi: 10.1016/j.postharvbio.2019.111095

[ref62] Pimentel R. B. Souza D. P. Albuquerque P. M. Fernandes A. V. Santos A. S. Duvoisin S. Jr.. (2018). Variability and antifungal activity of volatile compounds from *Aniba rosaeodora* Ducke, harvested from Central Amazonia in two different seasons. Ind. Crop. Prod. 123, 1–9. doi: 10.1016/j.indcrop.2018.06.055

[ref63] Pitt J. I. (1979). The Genus *Penicillium* and its Teleomorphic states *Eupenicillium* and Talaromyces, United States: Academic Press Inc. Ltd.

[ref64] Prakash B. Kujur A. Yadav A. Kumar A. Singh P. P. Dubey N. K. (2018). Nanoencapsulation: An efficient technology to boost the antimicrobial potential of plant essential oils in food system. Food Control 89, 1–11. doi: 10.1016/j.foodcont.2018.01.018

[ref65] Prakash B. Singh P. Mishra P. K. Dubey N. K. (2012). Safety assessment of *Zanthoxylum alatum* Roxb. Essential oil, its antifungal, antiaflatoxin, antioxidant activity and efficacy as antimicrobial in preservation of *Piper nigrum* L. fruits. Int. J. Food Microbiol. 153, 183–191. doi: 10.1016/j.ijfoodmicro.2011.11.007, PMID: 22137251

[ref66] Rajkumar V. Gunasekaran C. Dharmaraj J. Chinnaraj P. Paul C. A. Kanithachristy I. (2020). Structural characterization of chitosan nanoparticle loaded with *Piper nigrum* essential oil for biological efficacy against the stored grain pest control. Pestic. Biochem. Physiol. 166:104566. doi: 10.1016/j.pestbp.2020.104566, PMID: 32448420

[ref67] Raper K. B. Fennel D. I. (1965). The Genus *Aspergillus*. Baltimore, USA: The Williams and Wikings Comp.

[ref68] Re R. Pellegrini N. Proteggente A. Pannala A. Yang M. Rice-Evans C. (1999). Antioxidant activity applying an improved ABTS radical cation decolorization assay. Free Radic. Biol. Med. 26, 1231–1237. doi: 10.1016/S0891-5849(98)00315-3, PMID: 10381194

[ref70] Sajomsang W. Gonil P. Saesoo S. Ovatlarnporn C. (2012). Antifungal property of quaternized chitosan and its derivatives. Int. J. Biol. Macromol. 50, 263–269. doi: 10.1016/j.ijbiomac.2011.11.004, PMID: 22100980

[ref71] Sampaio L. D. F. S. Maia J. G. S. de Parijós A. M. de Souza R. Z. Barata L. E. S. (2012). Linalool from rosewood (*Aniba rosaeodora* Ducke) oil inhibits adenylate cyclase in the retina, contributing to understanding its biological activity. Phytother. Res. 26, 73–77. doi: 10.1002/ptr.3518, PMID: 21544884

[ref72] Singh V. K. Das S. Dwivedy A. K. Rathore R. Dubey N. K. (2019). Assessment of chemically characterized nanoencapuslated *Ocimum sanctum* essential oil against aflatoxigenic fungi contaminating herbal raw materials and its novel mode of action as methyglyoxal inhibitor. Postharvest Biol. Technol. 153, 87–95. doi: 10.1016/j.postharvbio.2019.03.022

[ref73] Singh B. K. Tiwari S. Dubey N. K. (2021). Essential oils and their nanoformulations as green preservatives to boost food safety against mycotoxin contamination of food commodities: a review. J. Sci. Food Agric. 101, 4879–4890. doi: 10.1002/jsfa.11255, PMID: 33852733

[ref74] Soltanzadeh M. Peighambardoust S. H. Ghanbarzadeh B. Mohammadi M. Lorenzo J. M. (2021). Chitosan nanoparticles encapsulating lemongrass (*Cymbopogon commutatus*) essential oil: physicochemical, structural, antimicrobial and *in-vitro* release properties. Int. J. Biol. Macromol. 192, 1084–1097. doi: 10.1016/j.ijbiomac.2021.10.070, PMID: 34673101

[ref75] Sruthi N. U. Rao P. S. (2021). Effect of processing on storage stability of millet flour: A review. Trends Food Sci. Technol. 112, 58–74. doi: 10.1016/j.tifs.2021.03.043

[ref76] Teles A. M. Silva-Silva J. V. Fernandes J. M. P. Calabrese K. D. S. Abreu-Silva A. L. Marinho S. C.. (2021). *Aniba rosaeodora* (Var. amazonica Ducke) essential oil: chemical composition, antibacterial, antioxidant and antitrypanosomal activity. Antibiotics 10, 24. doi: 10.3390/antibiotics10010024, PMID: 33396612 PMC7824638

[ref77] Timilsena Y. P. Adhikari R. Barrow C. J. Adhikari B. (2016). Microencapsulation of chia seed oil using chia seed protein isolated chia seed gum complex coacervates. Int. J. Biol. Macromol. 91, 347–357. doi: 10.1016/j.ijbiomac.2016.05.058, PMID: 27212219

[ref79] Tiwari S. Upadhyay N. Singh B. K. Singh V. K. Dubey N. K. (2022). Facile fabrication of Nanoformulated *Cinnamomum glaucescens* essential oil as a novel green strategy to boost potency Against food borne Fungi, Aflatoxin synthesis, and lipid oxidation. Food Bioprocess Technol. 15, 319–337. doi: 10.1007/s11947-021-02739-3

[ref80] Upadhyay N. Singh V. K. Dwivedy A. K. Chaudhari A. K. Dubey N. K. (2021). Assessment of nanoencapsulated *Cananga odorata* essential oil in chitosan nanopolymer as a green approach to boost the antifungal, antioxidant and in situ efficacy. Int. J. Biol. Macromol. 171, 480–490. doi: 10.1016/j.ijbiomac.2021.01.024, PMID: 33428956

[ref81] Upadhyay N. Singh V. K. Dwivedy A. K. Das S. Chaudhari A. K. Dubey N. K. (2018). *Cistus ladanifer* L. essential oil as a plant based preservative against molds infesting oil seeds, aflatoxin B_1_ secretion, oxidative deterioration and methylglyoxal biosynthesis. LWT 92, 395–403. doi: 10.1016/j.lwt.2018.02.040

[ref82] Vetriventhan M. Upadhyaya H. D. (2019). Variability for productivity and nutritional traits in germplasm of kodo millet, an underutilized nutrient-rich climate smart crop. Crop Sci. 59, 1095–1106. doi: 10.2135/cropsci2018.07.0450

[ref83] WorldCat, (2011). European Pharmacopoeia 7th edition, Council of Europe, Strasbourg, 1160–1161.

[ref84] Xu W. T. Peng X. L. Luo Y. B. Wang J. A. Guo X. Huang K. L. (2009). Physiological and biochemical responses of grapefruit seed extract dip on ‘Redglobe’grape. LWT 42, 471–476. doi: 10.1016/j.lwt.2008.09.002

[ref85] Xu D. Wei M. Peng S. Mo H. Huang L. Yao L.. (2021). Cuminaldehyde in cumin essential oils prevents the growth and aflatoxin B_1_ biosynthesis of *Aspergillus flavus* in peanuts. Food Control 125, 107985. doi: 10.1016/j.foodcont.2021.107985

[ref86] Yang E. Lee J. W. Chang P. S. Park I. K. (2021a). Development of chitosan-coated nanoemulsions of two sulfides present in onion (*Allium cepa*) essential oil and their nematicidal activities against the pine wood nematode, *Bursaphelenchus xylophilus*. Environ. Sci. Pollut. Res. 28, 69200–69209. doi: 10.1007/s11356-021-15451-8 34291413

[ref87] Yang S. Yan D. Li M. Li D. Zhang S. Fan G.. (2021b). Ergosterol depletion under bifonazole treatment induces cell membrane damage and triggers a ROS-mediated mitochondrial apoptosis in *Penicillium expansum*. Fungal Biol. 126, 1–10. doi: 10.1016/j.funbio.2021.09.002, PMID: 34930554

[ref88] Yoksan R. Jirawutthiwongchai J. Arpo K. (2010). Encapsulation of ascorbyl palmitate in chitosan nanoparticles by oil-in-water emulsion and ionic gelation processes. Colloids Surf. B: Biointerfaces 76, 292–297. doi: 10.1016/j.colsurfb.2009.11.007, PMID: 20004558

[ref90] Ziaee M. Moharramipour S. Mohsenifar A. (2014). Toxicity of *Carum copticum* essential oil-loaded nanogel against *Sitophilus granarius* and *Tribolium confusum*. J. Appl. Entomol. 138, 763–771. doi: 10.1111/jen.12133

